# Molecular docking and identification of G-protein-coupled receptor 120 (GPR120) agonists as SARS COVID-19 MPro inhibitors

**DOI:** 10.1186/s43141-022-00375-8

**Published:** 2022-07-18

**Authors:** Sellappan Mohan, Jayagopal Dharani, Ramanathan Natarajan, Arumugam Nagarajan

**Affiliations:** 1 Karpagam College of Pharmacy, Coimbatore, Tamil Nadu 641032 India; 2 Sri Sarada Niketan College for Women, Karur, Tamilnadu, India; 3 Saranathan College of Engineering, Panjappur, Tiruchirappalli, India

## Abstract

COVID-19 has become a pandemic, and any new drug for treating the disease could save millions of lives. Several drugs already in use for other diseases and medical conditions are repurposed for treating COVID-19 in an attempt to find treatment for the disease without spending research time on ADME TOX and other studies on side effects. In this exercise, the drugs repurposed are from antiviral, antibiotics, antiviral for HIV and HCV, anti-cancer, natural medicines, etc. Possible repurposing anti-diabetic GPR-120 agonists used as for SAR-CoV-2 is attempted in the study by carrying out docking of 68 GPR-120 agonists. Ten of these compounds were found to have docking scores −8.3 to −8.0, and the best docking score was observed for an arylsulfonamide and a biarylpropanoic acid belonging to GPR120 agonists previously evaluated for the treatment of type II diabetes. These GPR120 agonists could serve as start point for novel inhibitors for the discovery of drugs to treat COVID-19.

**Supplementary Information:**

The online version contains supplementary material available at 10.1186/s43141-022-00375-8.

## Background

After suffering from a devastating spell of COVID-19, the world is slowly limping back to normalcy. This is one of the pandemics that has a better public awareness owing to the Internet and social media. As per the data reported to WHO Globally, as of 14 October 2021, there have been 239,007,759 confirmed cases of COVID-19, including 4,871,841 deaths. As of 13 October 2021, a total of 6,471,051,151 vaccine doses have been administered (https://covid19.who.int/ accessed on October 15, 2021). The world is undergoing the largest vaccination program to guard the people from any further spells of the deadly virus.

In addition to the vaccines, the pharmaceutical companies and the scientists in various organization are trying to develop drugs to combat the SARS CoV-2. There are several targets that could be explored to develop new drugs for COVID-19 [1]. The therapeutic targets include both structural and non-structural proteins [2]. Some of the targets considered to develop inhibitors are as follows:

- Spike protein (S-protein) [3–5]
- Angiotensin-converting enzyme-2 (ACE-2) [6, 7]
- Human proteases: Transmembrane protease, serine 2 (TMPRSS2) [7, 8], Furin [9], Papain like protease-2 (PLpro) [10–12] 3-chymotrypsin like protease (3-CLpro) or the main protease M^Pro^ [13]
- Viral proteases (RNA-dependent RNA-polymerase (RdRp) [14]

One of the steps taken by the scientific community to combat the pandemic was to repurpose drugs already known and in use. This provides a shortcut and reduces the considerable amount of time spent on ADME Tox studies and the burden on assessing the new drug molecule’s therapeutic efficacy, side effects, and risks. Several small molecules were considered [15]. The repurposed drugs are usually broad-spectrum antivirals that fall under the two therapeutic classes namely, protease inhibitor and nucleosides. Among the repurposed drugs favipiravir, remdesivir, molnupiravir, galidesirvir, sobosbivir, and azivudine are examples of nucleosides while boceprevir, narlaprevir, simeprevir, and calpain inhibitors belong to protease inhibitors.

The race in finding an antiviral for COVID-19 was given momentum by computer-aided drug design approach, especially with the aid of docking software such as Auto Dock and Schrödinger. Of the several targets mentioned above, the main protease (M^Pro^) has been the most explored for the development of inhibitors. One of the main reasons for exploring the M^Pro^ inhibitors is its important role played in the in the replication and transcription of SARS CoV-2 [16]. The main protease (M^Pro^) is one of the proteins encoded in SARS-CoV-2 genome and is a dimer of cystine protease. This is called the 3-chymotrypsin-like protease (3-CLpro). M^Pro^ presents a highly conserved active site in several coronaviruses, such as SARS-CoV and MERS-CoV. M^Pro^ plays an important role in the cleavage of precursor polyproteins translated from viral RNA, and no other human protease does have a similar cleavage specificity. This makes M^Pro^ an attractive target for developing inhibitors, and the inhibitor may thus be non-toxic.

The development of inhibitors targeting the main protease appears to have not left any stone unturned and these repurposed molecules may be grouped into (1) inhibitors of other CoV, (2) antiviral therapeutics of human immunodeficiency virus (HIV), (3) anti-viral that are being used in hepatitis C virus (HCV), (4) antimalarial and other antivirals for influenza, (5) anti-bacterial, (6) anti-cancer drugs, (7) Traditional Chinese medicines, and (8) chemicals in traditional spices and other natural compounds from marine origin.

**Fig. 1:**
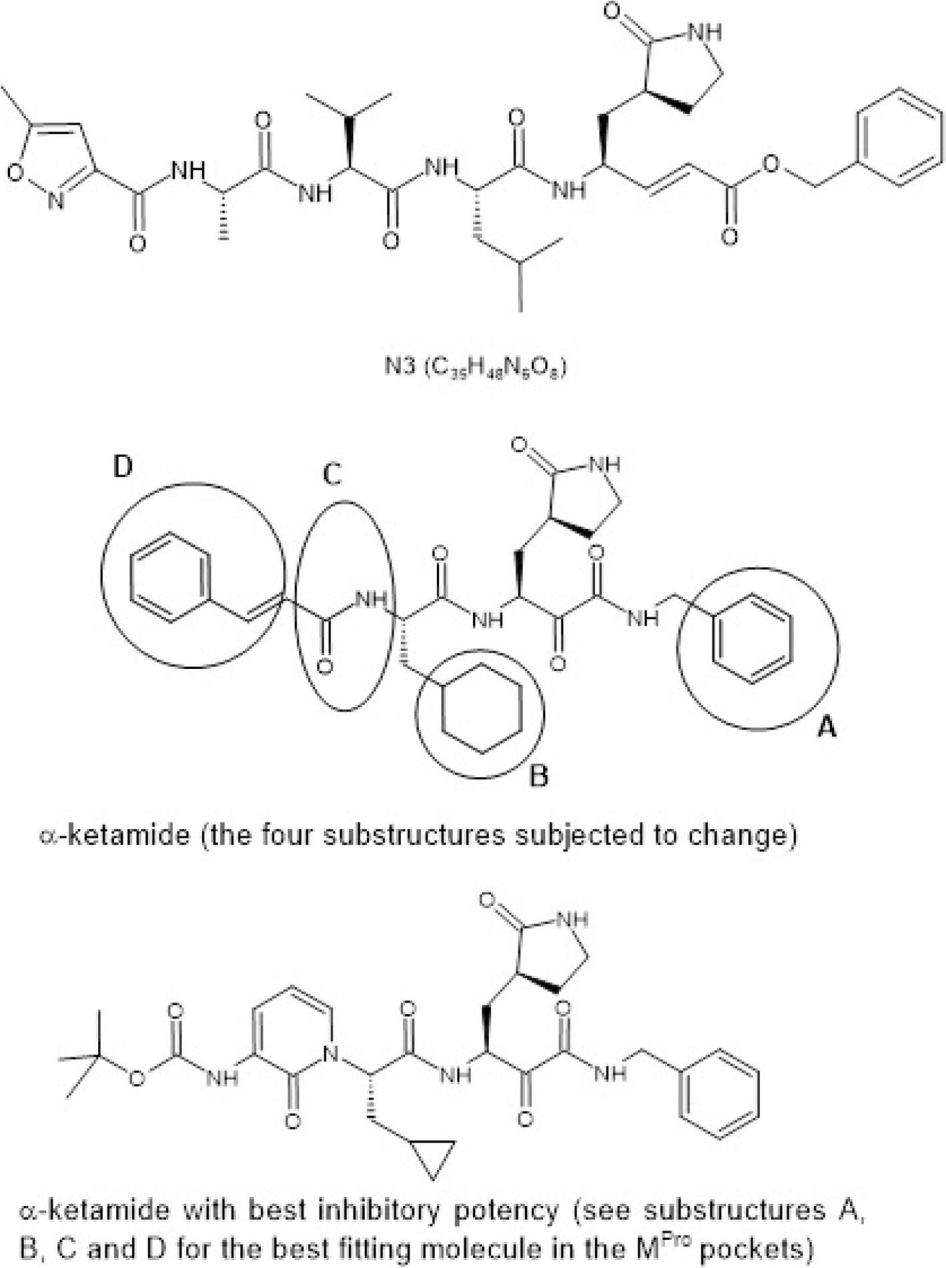
Structure of N3, α-ketamide with sub-structures marked

Based on the action of the main protease, Yang et al. has designed several inhibitors in 2005. The authors found molecule N3 (number assigned by Yang et al. [17]) (see Fig. 1) as the most potent inhibitor of CoV. This molecule was studied by Jin et al. [13] for SARS CoV-2 and from the results of docking study the authors proposed that N3 binds in 3CL^Pro^ binding pockets in an irreversible manner, and they thus exhibited good inhibitory potency. The 3CL^Pro^ complex withN3 molecule was used to identify new inhibitors and one such molecule is ebselen [13, 18]. Ebselen, a drug used for the treatment of stroke containing a selenium atom is repurposed for SARS-CoV-2. Crystal structure of M^Pro^ without any ligand bound to the protein was reported by Zhang et al. [19] and Zhang et al. [20] studied the binding affinity of alpha-ketamide to 3CL^Pro^ and identified three binding pockets in the protein. By varying the four substructures (marked as A, B, C, & D in Fig. 1), they obtained the best fitting into the protein pockets from the inhibitory potency (Fig 1).

Calligari et al. [21] investigated thirteen proteinase inhibitors that are used as antiviral for human immunodeficiency virus (HIV) and hepatitis C virus (HCV). The ten anti-HIV drugs are saquinavir, indinavir, tipranavir, ritonavir, lopinavir, atazanavir, nelfinavir, amprenavir, darunavir, and fosamprenavir while the three anti-HCV aresimeprevir, faldaprevir, and asunaprevir. Among these simeprevir was found to have the highest docking score. Lopinavir/ritonavir, coformulation is sold under the brand name Kaletra as an antiretroviral medication for the treatment and prevention of HIV/AIDS. Repurposing of Kaletra for SARS CoV-2 was found to be effective. Nutho et al. [22] could explain the inhibitory efficacy of Kaletra based on the docking studies of lopinavir and ritonavir with 3CL^Pro^. Chang et al. [23] showed that indinavir binds with 3CL^Pro^ stronger than lopinavir and ritonavir and Calligari et al. [21] had also inferred this in their study. Nelfinavir was identified to be a potential inhibitor for CoV-2 from a docking that used 1903 candidates [24]. These authors went on to determine the inhibitory potency of nelfinavir [25]. Atazanavir the HIV antiviral was found to be a potential inhibitor of 3CLPro [26], and its ability to inhibit SAR CoV-2 Vero cells was studied by Fintelmen_Rodrigues et al. [27].

In addition to the three HCV drugs mentioned above, ledipasvir and velpatsavir were reported by Chen in 2020 [28]. Li et al. [29] ended up with four molecules namely, prulifloxacin, bictegravir, nelfinavir, and tegobuvir by high through put screening of 8000 clinical drug libraries based on the binding affinity with M^Pro^. Khan et al. [30] screened 123 antiviral drugs to identify inhibitors of 3CLPro as well as 2′-O-MTase (2′-O-ribose methyltransferase). Paritaprevir and Raltegravir were found to have high binding affinity for 3CL^Pro^.

Talluri [31] carried out virtual screening of several clinically approved antiviral and the crystal structure of M^Pro^ (PDB if 6LU7) and found saquinavir and beclabuvir as the best protease inhibitor candidate SARS CoV-2 among the compounds studied by them. Other anti-viral drugs that had been tested for repurposing by molecular docking and virtual screening include oseltamivir [32] and zanamivir [33].

Some of the antibiotics that have been identified to be effective based on computer-aided virtual screening are the quinoline antibiotic prulifloxacin [29], tetracycline antibiotics eravacycline [34], and the polypeptide antibiotic colistin [35]. Non-steroidal anti-inflammatory drugs (NSAID) were also repurposed as potential inhibitors of MPro [36] by docking studies. In a similar study on NSAIDs, Gimeno et al. [37] identified Perampanel, Carprofen, Celecoxib, Alprazolam, Trovafloxacin, Sarafloxacin, and ethyl biscoumacetate as possible inhibitors of M^Pro^ by docking studies. The two compounds namely, Carprofen, a NSAID no longer use in human medicine but used for veterinary purpose, and Celecoxib another NSAID and a COX-2 inhibitor, were subjected to in vitro testing at 50 μM, and they showed 3.97% and 11.90% M^Pro^ inhibition, respectively.

*Dipyridamole* (brand name Persantine) is a platelet inhibitor and is used to prevent blood clots after heart surgery was repurposed for CoV-2 by Liu et al. [38] and the inhibitory potency (IC_50_) was studied targeting 3CL^Pro^. Odhar et al. [39] studied the drug molecules from US-FDA-approved drugs library from ZINC 5 database, and from their docking on to the M^Pro^ (PDB id 6LU7), they identified ten hits that included drug that are used for cancer, epilepsy, and insomnia. The top ten hits based on the docking score are Perampanel (epilepsy), conivaptan (hyponatremia), sonidegib (basal-cell carcinoma), azelastine (allergy), idelalisib (leukemia and lymphoma), suvorexant (insomnia) olaparib (ovarian, breast, and pancreatic cancers), ponatinib (leukemia), loxapine (schizophrenia), and tolvaptan (hyponatremia). Wang et al. [34] in the computational drug repurposing study identified carfilzomib (antineoplastic agent), valrubicin (chemotherapy drug), and elbasvir (antiviral for HCV) as inhibitors based on the docking with M^Pro^, in addition to the antibiotic eravacycline.

Traditional Chinese medicine (TCM) and traditional Indian medicines that fall under Ayurveda and Sidha were used as immune boosters to fight against COVID-19. Zhang et al. [19, 20] carried out docking studies of about 100 constituents of the Lung-toxin Dispelling Formula No. 1 (LDFN1) of TCM and found 22 of these chemicals are inhibitors of 3CL^Pro^. Of the several chemicals, baicalin and baicalein were found to have antiviral activities against 3CL^Pro^ [40] with EC_50_ values of 10.27 μM and 1.69 μM, respectively. Liu et al. [41] studied the inhibitory activity of the ethanol extract of the herbal plant *Scutellaria baicalensis* and its major component, baicalein. They found that the plant extract and the constituent baicalein inhibited SARS-CoV-2 3CL^pro^ activity in vitro with IC_50_ of 8.52 mg/mL and 0.39 mM, respectively. The replication of SARS-CoV-2 in Vero cells were inhibited with EC50s of 0.74 mg/ml and 2.9 mM, respectively. In their study on screening several natural compounds that are constituents of TCM, Zhang et al. [19] and Zhang et al. [20] identified betulinic acid, coumaroyltyramine, cryptotanshinone, desmethoxyreserpine, dihomo-γ-linolenic acid, kaempferol, lignan, *N*-cis-feruloyltyramine, quercetin, sugiol, and tanshinoneiia to inhibit 3CL^Pro^. Cherrak et al. [42] studied several glycosylated flavonoids by docking them on the M^Pro^ (6LU7) and identified quercetin-3-O-rhamnoside to have the highest binding affinity. Myricetin 3-rtinoside and rutin were also identified as potential inhibitors of 3CL^Pro^, and the binding affinities of these three compounds were greater than that of N3 with 3CL^Pro^. Shivanika et al. [43] carried out docking studies of several natural products that have been used as antiviral on to 6LU7 the 3CLPro protein structure and found theaflavin-3-3’-digallate, rutin, hypericin, robustaflavone, and (-)-solenolide as the compounds with highest binding energy. It might be noted that identification of rutin as a potential inhibitor is independently confirmed by two groups. Bhaliya and Shah [44] carried out docking studies of mono-carbonyl analogs of curcumin with 3CLPro and found one of the curcumin analogs was found to have potential to be used as an inhibitor. Joshi et al. [45] screened a library of ∼7100 molecules that comprises of flavonoids, glucosinolates, anti-tussive, anti-influenza, anti-viral, terpenes, terpenoids, alkaloids, and other compounds predicted as potential therapeutic candidates against M^Pro^. Molecules such as δ-viniferin, myricitrin, taiwanhomoflavone A, lactucopicrin 15-oxalate, nympholide A, afzelin, biorobin, hesperidin, and phyllaemblicin B were found to bind strongly with M^Pro^ and hence suggested as potential inhibitors. Andrographolide a natural compound from *Andrographis paniculata* was studied [46] via docking on to MPro, and the in silico studies on ADME and toxicity prediction were also carried out. The molecule was predicted to have good solubility. Ramaiah et al. [47] studied the binding of natural molecules that are present in Indian spices and curry against MPro (6LU7). A similar study identified [48] carnosol a natural molecule as an inhibitor by docking studies using the protein structure PDBID: 6Y84, MPro. Bioactive compounds in medicinal plants were screened as potential MPro inhibitors [49] and natural compounds such as kaempferol, quercetin, luteolin-7-glucoside, demethoxycurcumin, naringenin, apigenin-7-glucoside, oleuropein, curcumin, catechin, and epicatechin-gallate as potential molecules for further exploration.

According to the latest report of the pharmaceutical company Merck, molnupiravir pills are able to reduce the hospitalization and deaths of people affected by COVID-19 [50]. They reported the results of Phase 2a trial (ClinicalTrials.govNCT04405570) in which safety, tolerability, and antiviral efficacy of molnupiravir in the treatment of COVID-19. Merck applied on October 11, 2021, for US-FDA emergency use authorization for the molnupiravir based-oral antiviral pill for COVID-19. This will not stop the hunt for new inhibitors, and the search for new molecules will continue.

In one of the studies of repurposing drugs [51], virtually screened 1615 FDA approved drugs by docking each of them on to MPro and then refined the selection by employing molecular dynamics to identify nine compounds*.* The nine drugs selected as potential inhibitors vary from vasoconstrictor to microscopy dye. The potential inhibitors identified and their original use are:

1. Dihydroergotamine: vasoconstrictor
2. Midostaurin: treatment of acute myeloid leukemia
3. Ziprasidone: antipsychotic
4. Etoposide: antineoplastic
5. Apixaban: used to reduce the risk of stroke and blood clots
6. Fluorescein: a dye used in microscopy
7. Tadalafil: used to treat erectile dysfunction (ED), benign prostatic hyperplasia (BPH), and pulmonary arterial hypertension)
8. Rolapitant: used along with an antiemetic (anti-vomiting) agent in adults for the prevention of delayed nausea and vomiting associated with initial and repeat courses of emetogenic cancer chemotherapy
9. Palbociclib: used to treat HR-positive and HER2-negative breast cancer

The above discussion indicates that repurposing of drugs belonging to different classes have been evaluated for CoV-2. Human GPR120 is a transmembrane protein, characterized by the interactions with the endogenous ligand linoleic acid and docosahexaenoic acid. Apart from the key role played by GPR 120 in diabetes, it is also involved in many other disease conditions, including cancer, inflammation, and central nervous system (CNS) disorders. GPR 120 presents itself in many metabolic pathways, and its pivotal role in controling obesity and diabetes is worth mentioning. Using gene knockdown studies, GPR120 has been shown to induce chemoresistance in breast cancer treatment with epirubicin and cisplatin-highlighting the relevance of GPR 120 antagonists for chemotherapy [52]. Further, Toelzer et al. recently identified a linoleic acid binding pocket in the SARS-CoV-2 spike protein which prompted us to look for alternate drugs for binding with *COVID-19 MPro.* The present study reports the results of docking studies carried out using G-protein-coupled receptor (GPR) agonists against the MPro to identify any potential inhibitor of SARS CoV-2.

## Methods

Auto Dock 4.2.6 was used to perform docking study. Chemical structures were drawn using Chemoffice 2002. Three-dimensional structures of proteins were downloaded from protein data bank (PDB id: 6LU7) (https://www.rcsb.org/).

### Protein preparation

**Fig. 2:**
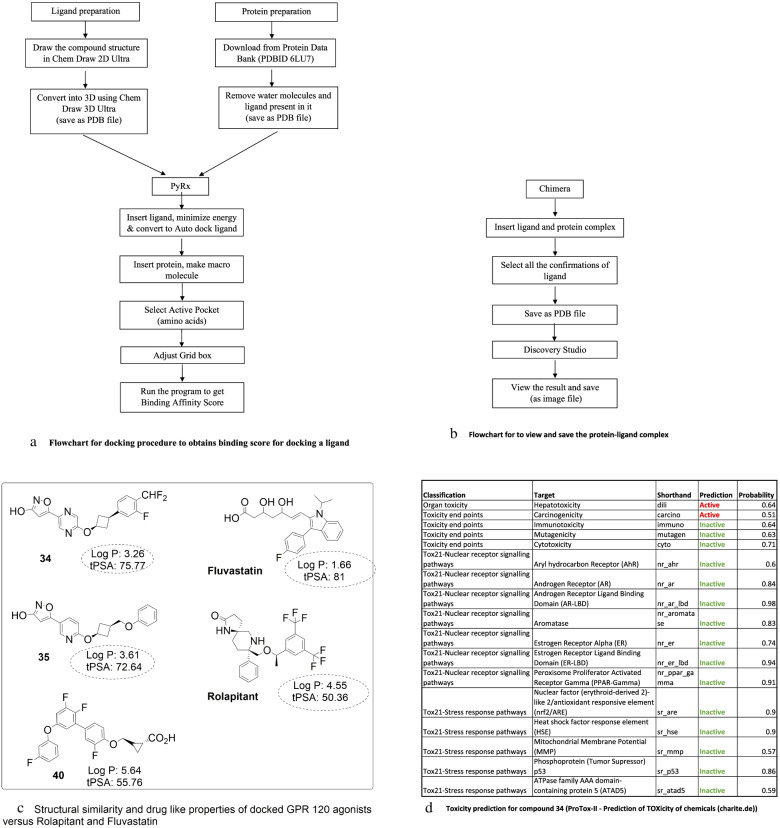
**a** Flowchart for docking procedure to obtain binding score for docking a ligand. **b** Flowchart for to view and save the protein-ligand complex. **c** Structural similarity and drug like properties of docked GPR 120 agonists versus Rolapitant and Fluvastatin*.* **d** *Toxicity prediction for compound* ***34*** (ProTox-II - Prediction of TOXicity of chemicals (charite.de))

The protein was prepared for docking process according to the standard protein preparation procedure integrated in Accelry’s Discovery Studio 4 which is shown in the flow chart (Fig. 2a).

Ligand 2D structures were drawn using ChemDraw Ultra 8.0 (ChemOffice 2002) and converted into 3D structure using chem3D Ultra 8.0. The 68 molecules were used as ligands, and each one of them was docked on to the crystal structure of M^Pro^ with PDB id 6LU7. The procedure for ligand preparation and docking is given as a flow chart in Fig*.* 2a*,* b. Docking scores were obtained to understand any inhibitory potential of the 68 GPR120 agonists.

Zhang and Macielag [53] discussed the patented GPR 120 agonists for the treatment of diabetes. They reviewed the therapeutic patents of ten different classes of compounds that amounted to 68 therapeutic molecules. The 68 GPR-120 agonists collected by the authors from different patents and journals are grouped into ten classes. The ten classes and the number assigned in this paper along with abbreviation are given below:

1. Natural GPR 120 agonists and early synthetic GPR 120 agonists (1–9)
2. Bi-aryl-based phenyl propionic acid derivatives as GPR 120 agonists (BiAr-PPA 10–14)
3. Cycloalkenyl and heterocycloalkenyl-based phenyl propionic acid derivatives (CycA_Hcyc--PPA 15–23)
4. Dihydrobenzofuran derivatives (Metabolex) and benzo-fused heterocyclic derivatives (Metabolex 24 and Jansesen 25–27)
5. Chemcial scaffolds claimed by Merck (Merck 28–35)
6. Various carboxylic acid scaffolds claimed by Bristol-Meyers Squibb (BMS 36–45)
7. Patented structures by Piramal Enterprises Limited (PEL 46–52
8. Other carboxylic acid-based GPR 120 agonists (Calden (53–54; LG 55–59; Ajinamoto 60; DOMPE 61–62)
9. Non-acid-containing structures claimed by AXXAM (AXXAM 63-65)
10. GPR120 agonists/antagonists in the peer-reviewed journals (GSK 65; U of B 66; GSK 67; Ch Pharm U 68).

Different classes of these 68 molecules are patented as GPR120 agonists to treat type-2 diabetes by various pharmaceutical companies namely, Janssen Pharmaceutica NV, Merck Sharp & Dohme Corp., Bristol Myers Squibb, Piramal Enterprises Limited, Caldan Therapeutics Limited, LG Life Sciences Ltd., Ajinomoto Company, Accepted Manuscript Inc., Dompe’ Farmaceutici S.P.A., and AXXAM S. P. A. Structures of these compounds with their abbreviated id number used in this paper are given in Table 1.

**Table 1:**
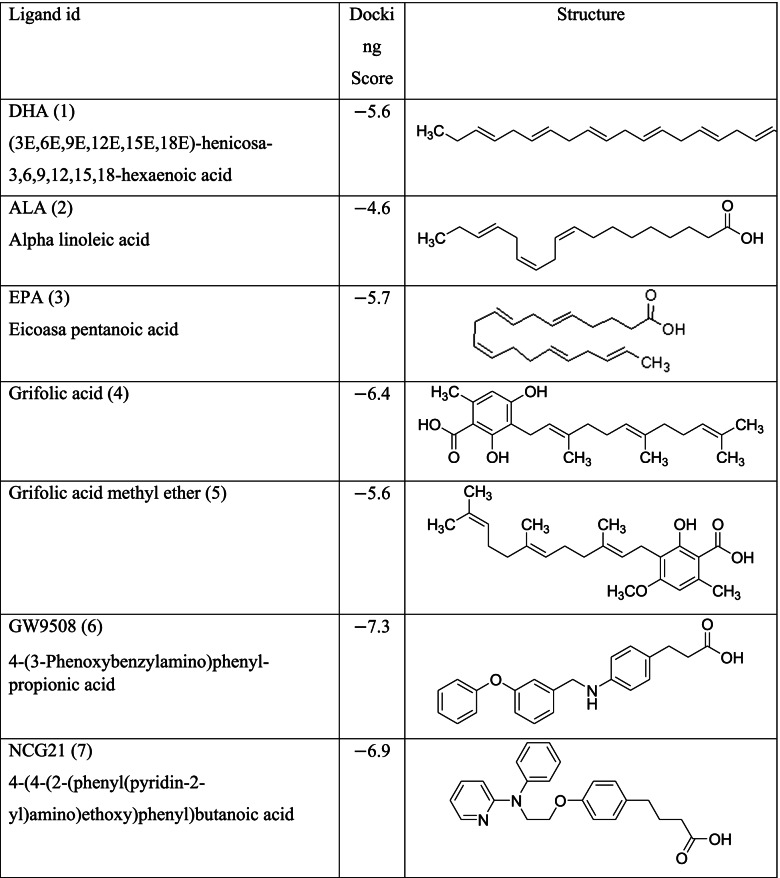

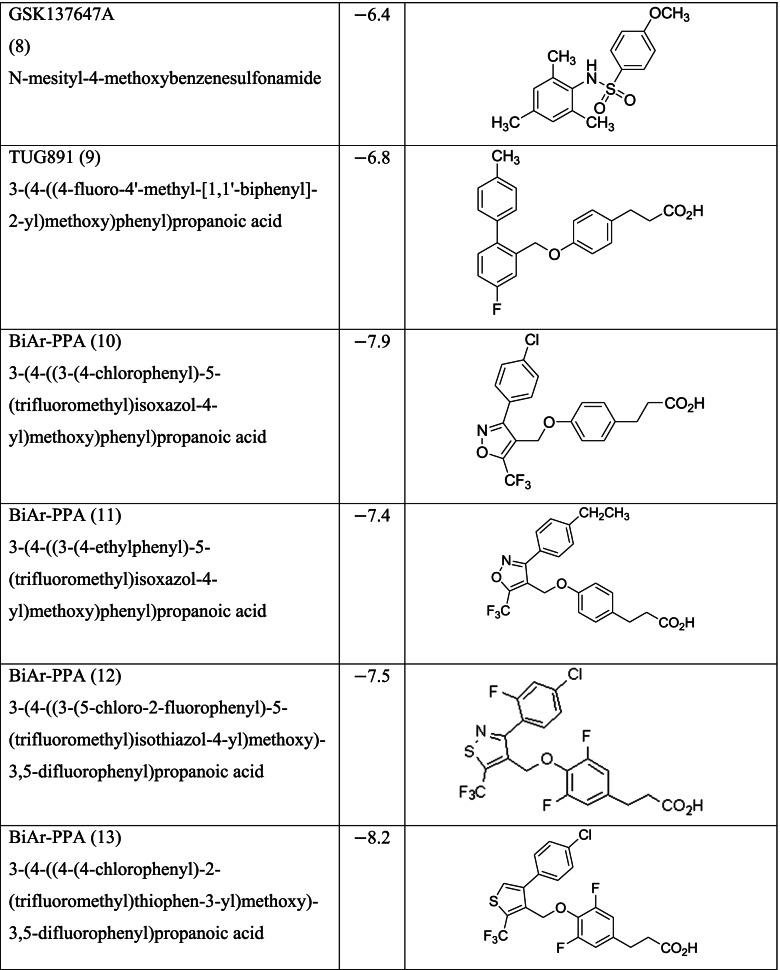

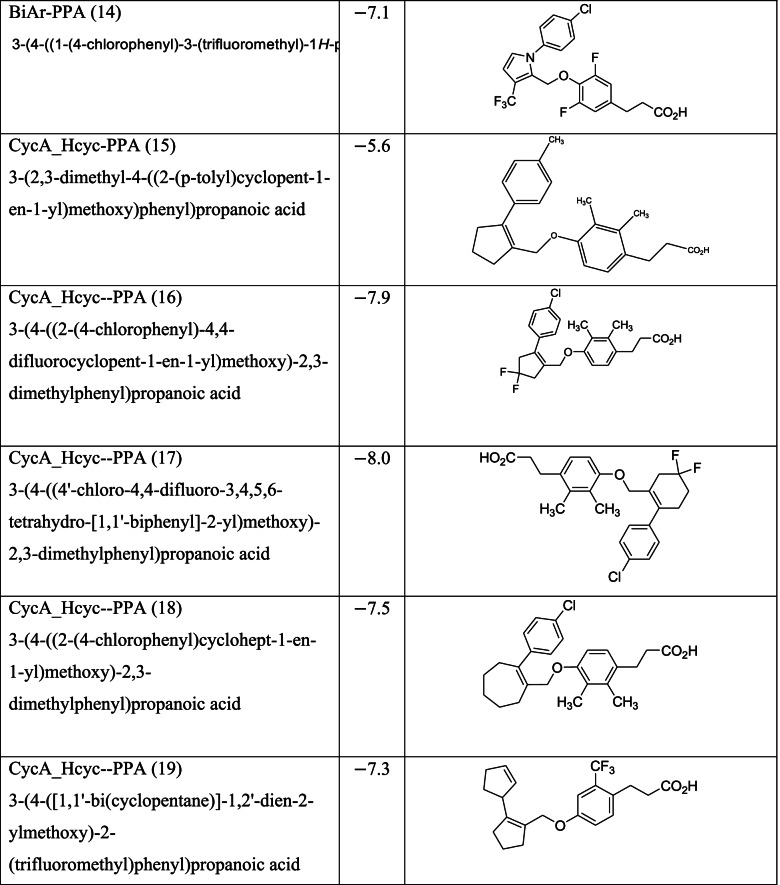

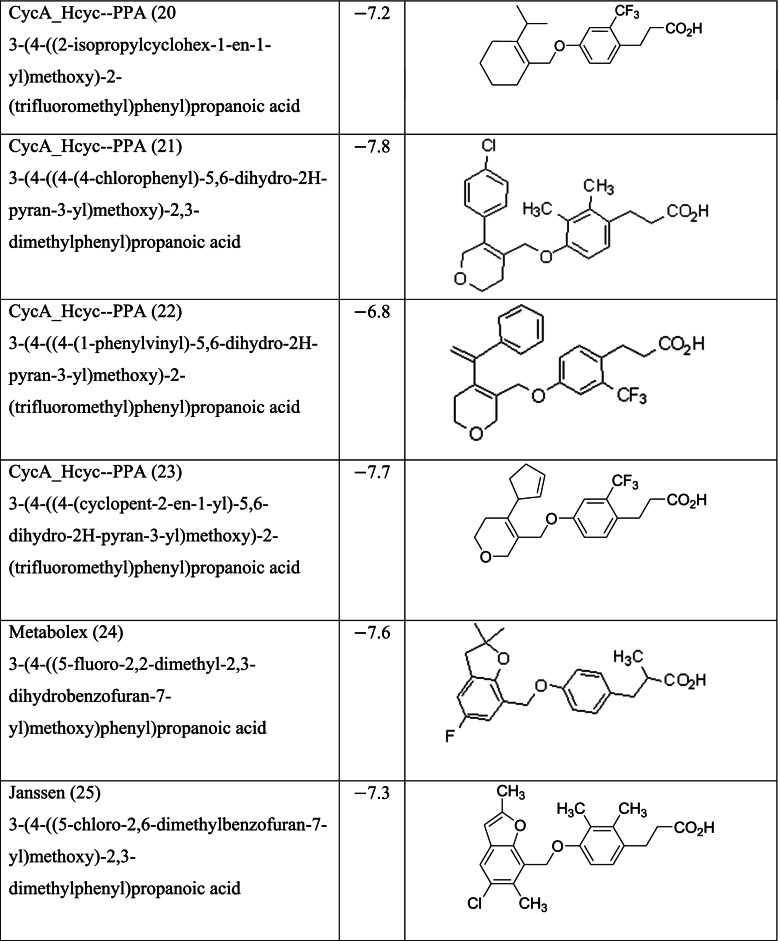

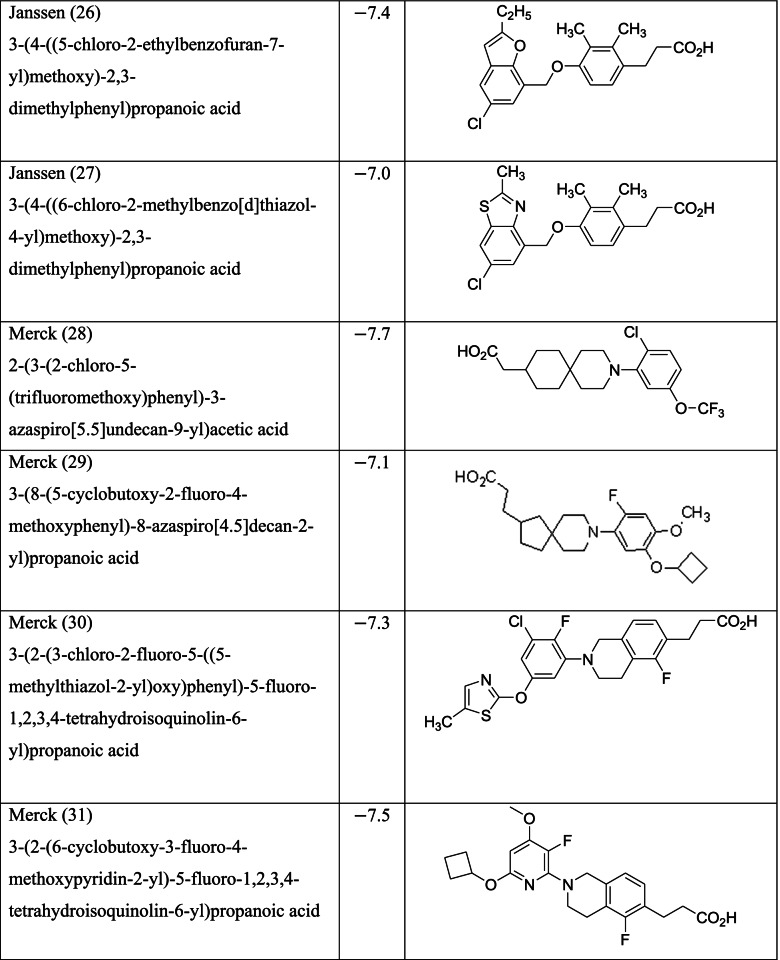

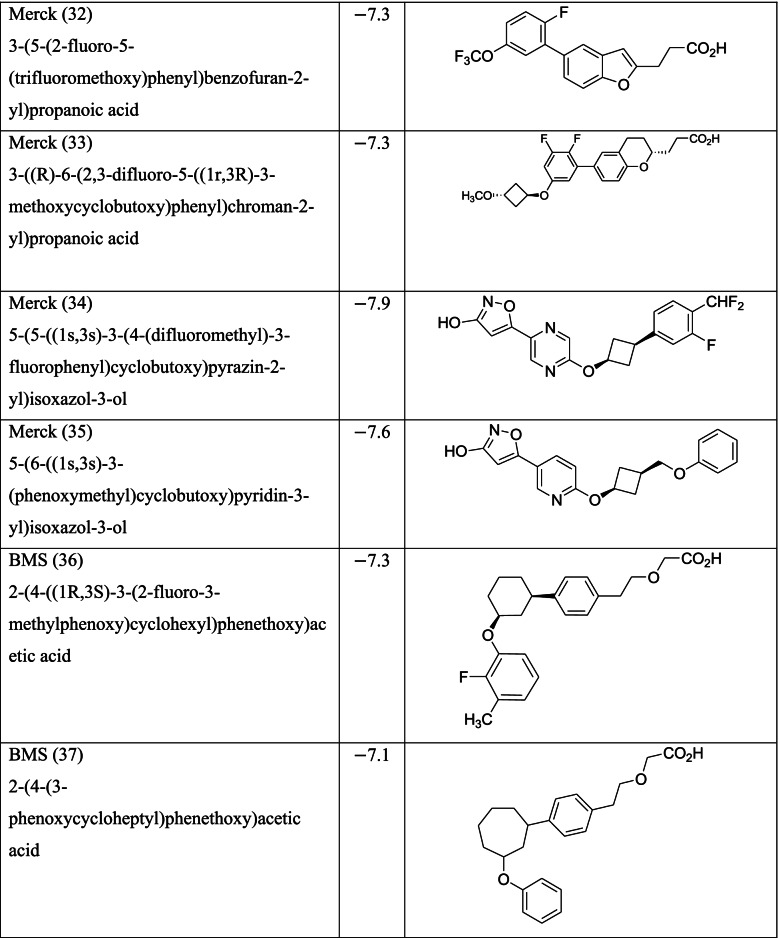

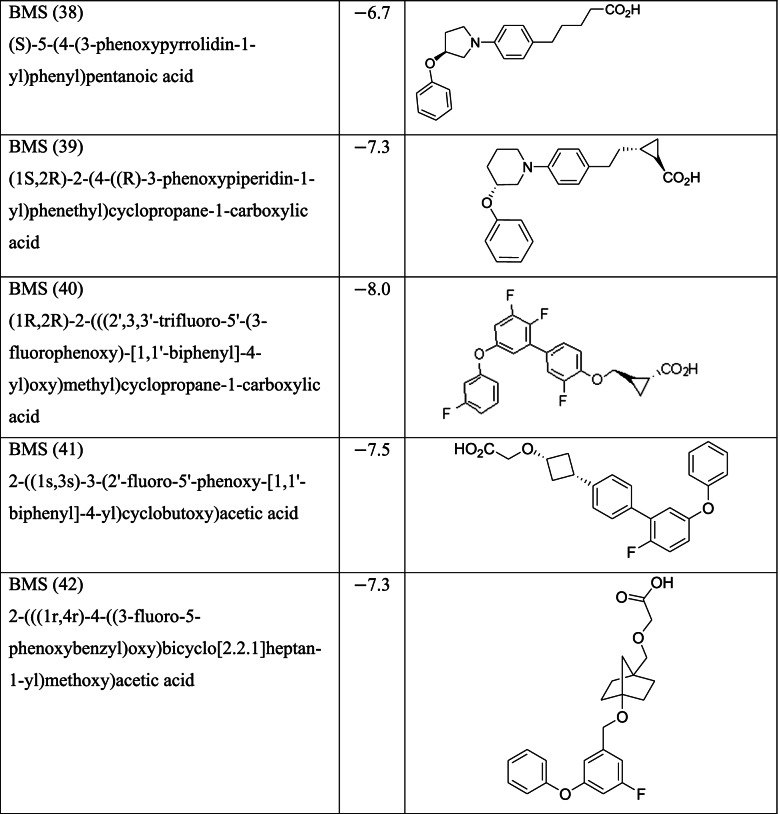

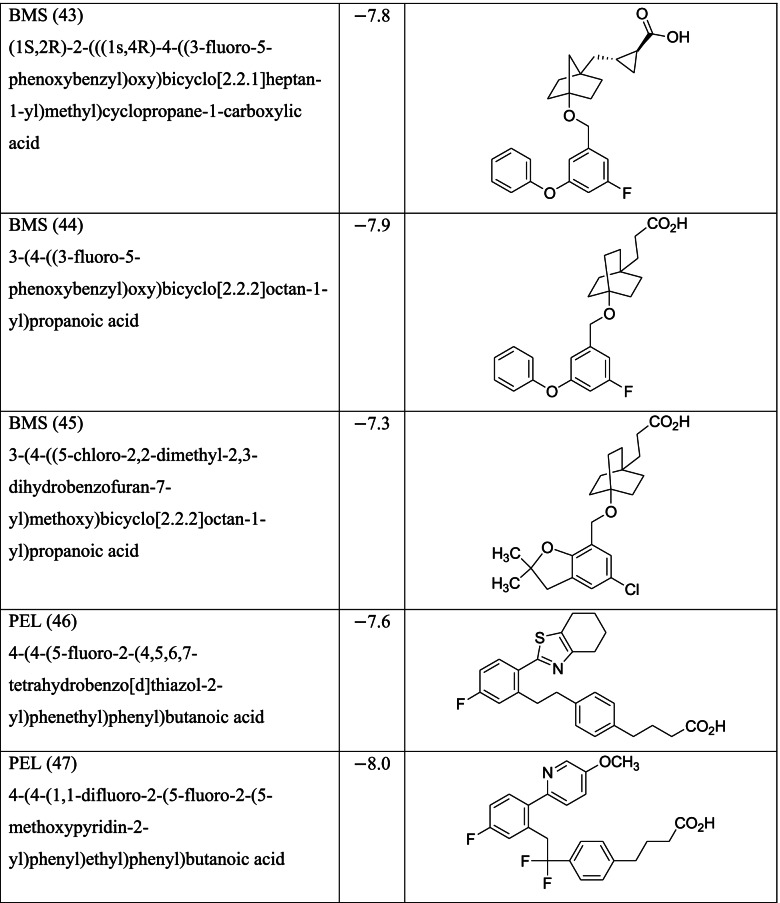

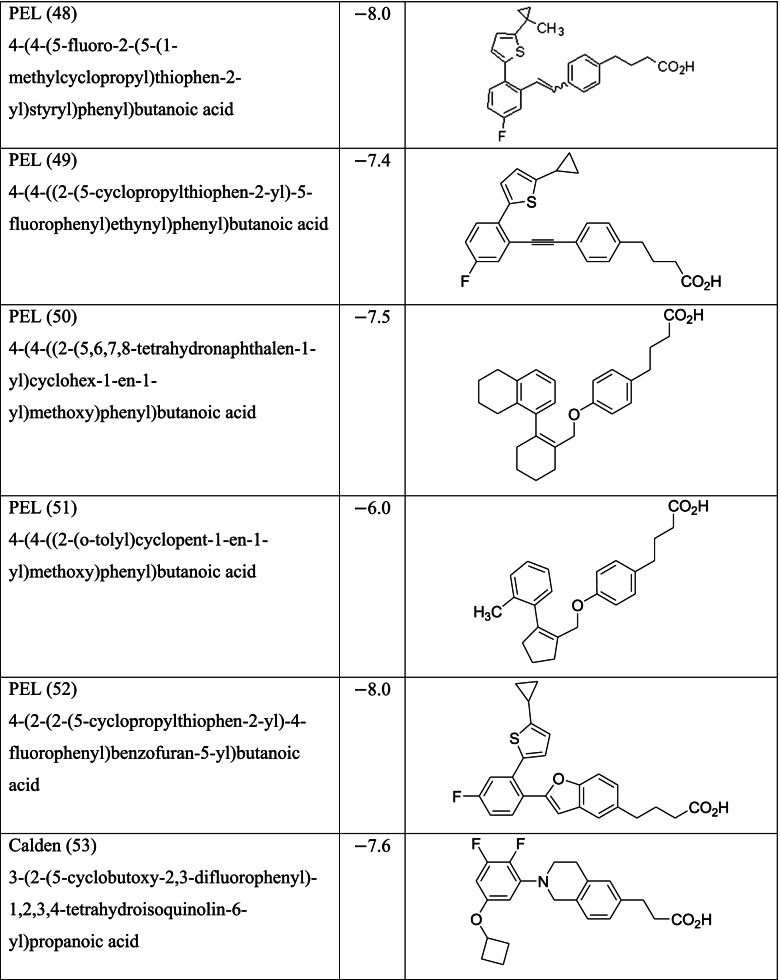

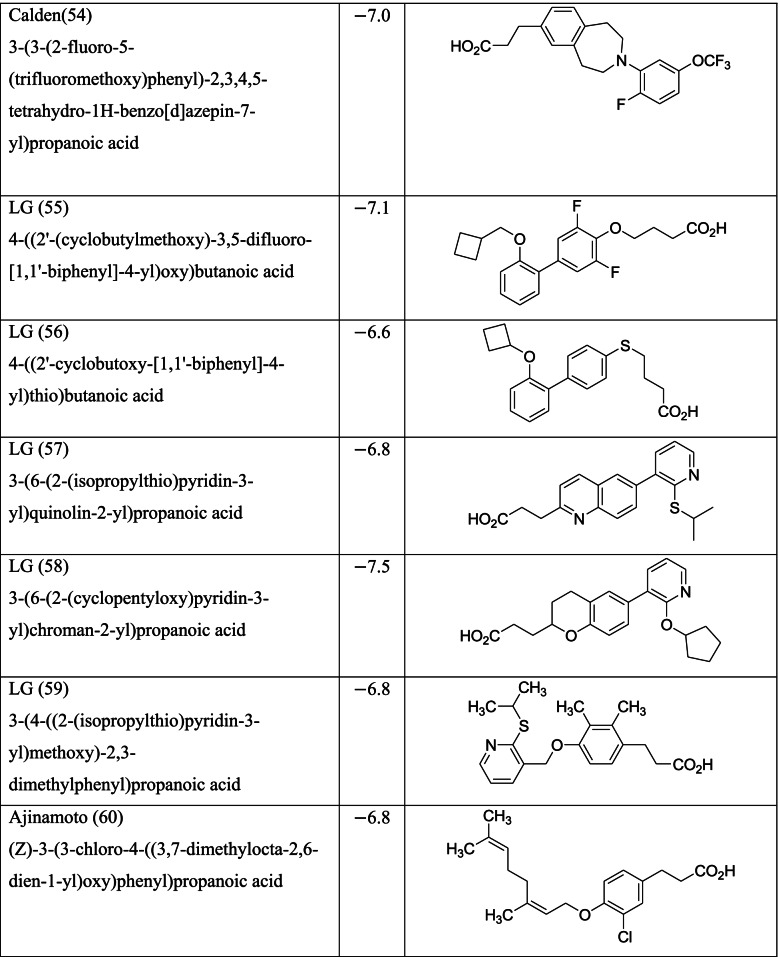

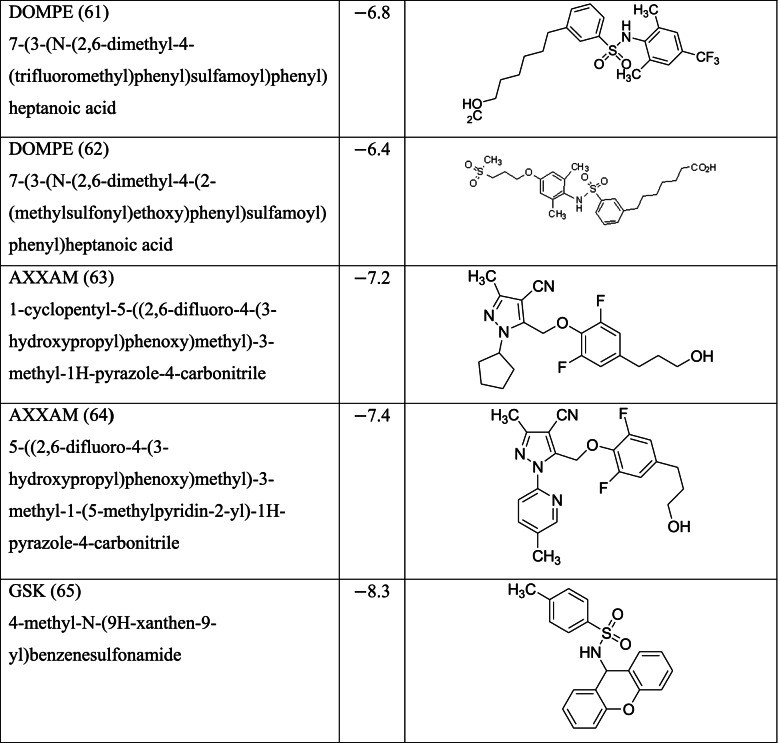

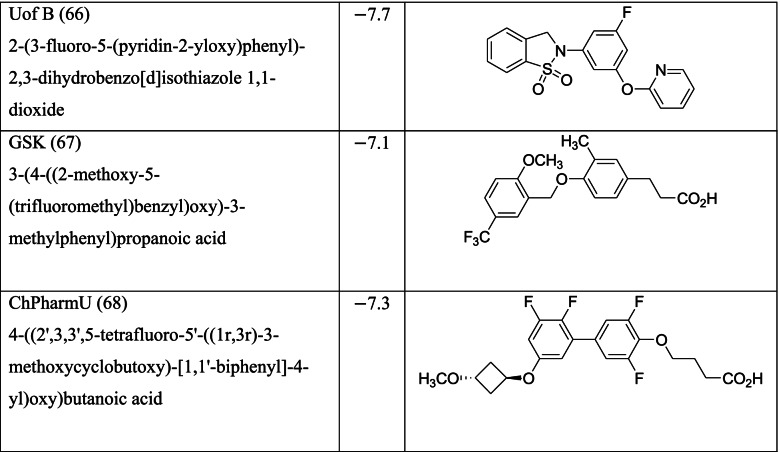
GPR120 agonists, ligand ids (used in this paper), docking scores, and molecular structures

## Results and discussions

**Fig. 3:**
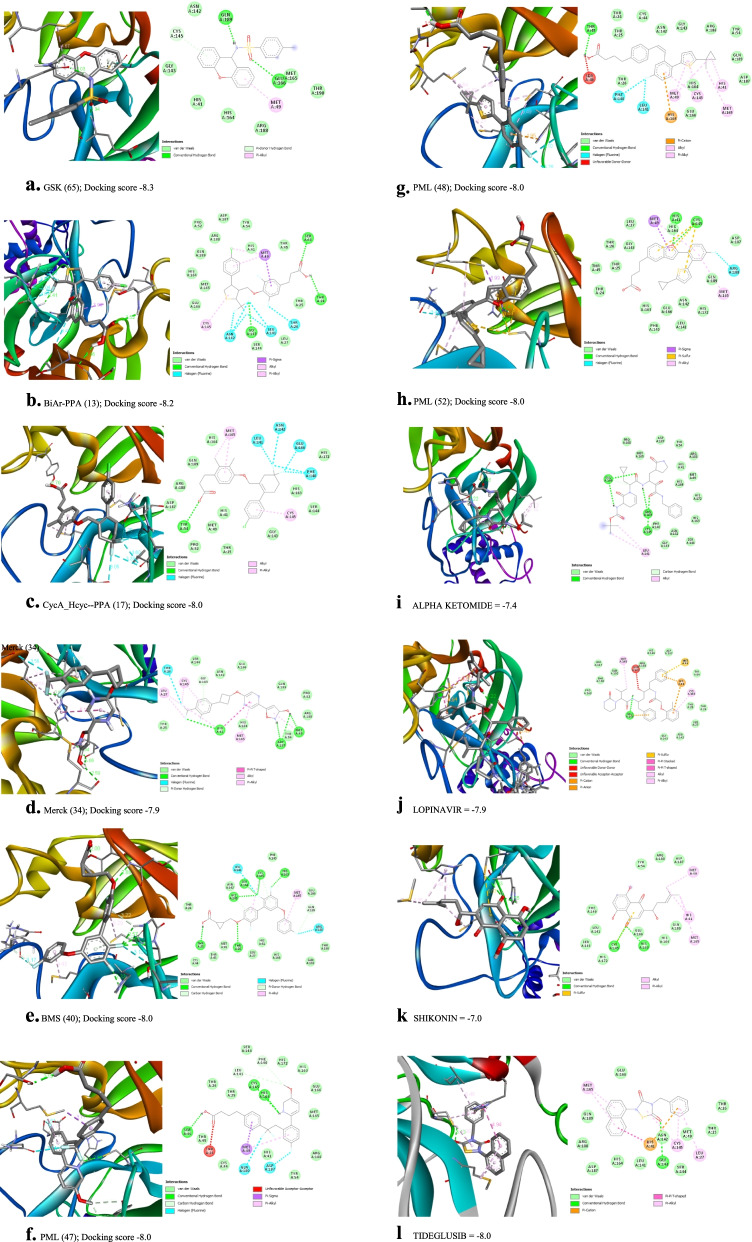
**a** GSK (65); Docking score −8.3. **b** BiAr-PPA (13); Docking score −8.2. **c** CycA_Hcyc--PPA (17); Docking score −8.0. **d** Merck (34); Docking score −7.9. **e** BMS (40); Docking score −8.0. **f.** PML (47); Docking score −8.0. **g** PML (48); Docking score −8.0. **h** PML (52); Docking score −8.0. Docking of the ligands onto MPro (6LU7). Docking images of ligands with docking score ≥ −7.9 are given (for other ligands please see Supplementary Information); **i** ALPHA KETOMIDE = -7.4; **j** LOPINAVIR = -7.9; **k** SHIKONIN = -7.0; **l** TIDEGLUSIB = -8.0

Binding scores for each of the 68 ligands are listed in Table 1 along with their molecular structures. Docking images each of the ligands in MPro (PDB ID 6LU7) are given in the Supplementary material while the ligands with best scores (≥ −8.0) are given in Fig. 3. The type of docking interaction for these ligands are presented in Table 2. Some of the ligands are having a docking score of ≥ −8.0. The lowest value is −8.3 for the molecule with id GSK (65). This is a sulfonamide patented by GlaxoSmithKline as a selective antagonist against free fatty acid 4 (FFA4/GPR120) [54, 55] and to be used with the agonist GSK 137647A which is also a sulfonamide (id in this paper GSK137647A(8)). The compound with binding score −8.2 is a biaryl-based phenylpropanoic acid (13)) [56] patented by Janssen Pharmaceutica [57]. The compound 17 with binding score −8.0 is also phenylpropanoic acid derivative namely, cyclohexenyl-based phenyl propionic acid [58]. Three of the other compounds 47, 48, and 52, with binding score −8.0 are patented by Piramal Enterprises Limited as GPR120 agonists [47, 59–61] and phenylbutanoic acid with biarylsubstituent wherein one of the aryl groups is a heterocyclic or fused heterocyclic system. Cyclopropane carboxylic acid derivative with a phnoxybiphenyl substituent **40** is also found to have a binding score 8.0. This molecule is patented by Bristol-Meyers Squibb Company [62] as GPR120 modulators useful for treatment of diabetes and related diseases.

**Table 2:** Docking interactions for the ligands with docking score ≥ −8.0

Ligand id	Docking score	Docking details		
		Conventional H-bond	Alkyl and pi-alkyl	others
GSK (65)	−8.3	GLN:189, GLU:166	MET:49	CYS:145 (Pi-donor hydrogen bond)
BiAr-PPA (13)	−8.2	THR:24, SER:46, GLY:143	MET:49, CYS:145	MET:49 (Pi-sigma) THR:26, LEU:141, ASN:142 (Halogen)
CycA_Hcyc--PPA (17)	−8.0	TYR:54	CYS:145, MET:165	PHE:140, LEU:141, ASN:142, GLU:166 (Halogen)
Merck (34)	−7.9	Asp 187, Tyr 54, His 164, His 41	Cys 145, Leu 27	
BMS (40)	−8.0	THR:25,26, GLY:143, SER:144, CYS:145, HIS:163 GLN:189, GLU:166	CYS:145, MET:165	GLN:189, CYS:145 (carbon hydrogen bond); GLY:143 (Pi-donor hydrogen bond); LEU:141, ARG:188 (Halogen)
PEL (47)	−8.0	SER:46, CYS:145, HIS:164	MET:49	THR:24 (unfavorable acceptor-acceptor); MET:49 (Pi-sigma) LEU:141, PHE:140 (carbon hydrogen bond); GLN:189, ASP:187 (Halogen)
PEL (48)	−8.0	THR:45	HIS:41, MET:49, CYS:145, MET:165	HIS:163 (Pi-cation); SER:46 (unfavorable donor-donor); PHE:140, LEU:141 (Halogen)
PEL (52)	−8.0	HIS:41, CYS:145	MET:49, MET:165	MET:49 (Pi-sigma); CYS:145 (Pi-sulfur); ARG:188 (Halogen)

Based on the binding score of the top 10 compounds investigated here, their role in blocking the binding site through Glu 166 and Cys 145 could be considered relevant for their potential role as novel ligands for Sars-COVID-19 virus protein. The observation that Remdesivir, Nelfinavir, and other antiviral compounds show similar interaction support our inference [63–65]. Additional support for such a claim has been found in the paper describing docking study of metocurine with M-Pro 6LUZ that indicate the drug occupies the binding site [66]. The important residues observed in the docking study of our GPR120 agonists as well as the above molecules studied by others including that of chlorquine [61] highlight the role of NH, COOH groups in manifesting pi bond formation with Glu 166 and aromatic pi interaction with Cys 145, respectively. Dock score for the reference compounds (Fig. 3i–l) evaluated along with the GPR120 agonists ALPHA KETOMIDE (−7.4), LOPINAVIR (*−*7.9), SHIKONIN (*−*7.0), and TIDEGLUSIB (*−*8.0) indicate the interactions with Glu 166 and Cys 145 are present in these drugs also.

Compounds 13, 16, 17, 44, and 50 identified with high dock score are hydrophobic compounds having thiophenyl, cyclopentenyl, cyclohexenyl, norbornyl, and cyclopentenyl groups along with a phenylpropanoic acid function. Compound 40 is a phenoxyphenyl ether having a cyclopropropane carboxylic acid group while 34 has an isoxazolyl and pryrimidine compound with difluoromethane function. Compound 65 is a tricyclic compound having arylsulfonamide function. Compounds 13 and 52 are highly lipophilic having a log *P* value of 7.1 and 6.25, respectively, that might require vigorous optimization to make them orally available. On the other hand, linoleic acid has a log P value of 5.65 while that of LOPINAVIR is 4.56 and the value is 4.86 for TIDEGLUSIB*.* Hosseini et al. screened several classes of drugs and identified inhibitors for SARS-CoV-2 MPro and highlighted H bond interactions with Thr 26, Phe 140, Gly 143, Glu 166, and Gln 189 in addition to pi stacking interaction through His 140 as key contributors for receptor binding [67]. Our GPR 120 agonists, 40, 47, and 65 revealed H bonding interaction with Glu 189 in the docking against MPro while hydrophobic interactions with His 41, Met 165, and Glu 166 were shown by compounds 40, 48, 52, and 65. Fluvastatin on the other hand was found to interact with Thr 26 and Gly 143 by Maryam et al., and a similar interaction was observed in our compound 40, which also interacted with Glu 166 and Cys 145. Compounds 65 and 34 have a log P value of 4.45 and 3.26, respectively, indicating that they might have a good oral bioavailability, although they require further optimization. The comparison of topological polar surface area (TPSA) of the compounds evaluated show that only compound 34 has a TPSA value above 75, whereas the reference compounds have TPSA of 120 for lopinavir, a-ketoamide has a value of 113. This suggests that compounds 34 and 40 could be used as start points, and further optimizations could result in finding a drug for Sars-COVID-19. Compound 34 possesses a hydroxyl isoxazole group that would mimic COOH function and also the presence of polar heterocyclic ring providing it an ideal choice to improve its physicochemical characteristics. The compound **34** is in fact well anchored through H-bonded interactions, Pi interactions as shown in Fig. 3d. Similar to compound 40, having a cyclopropane carboxylic acid function could be optimized further to refine its log P to make this eligible as a lead compound. Obviously the GPR 120 agonists, designed as agonists for free fatty acid receptors, have functional groups and lipophilic characters designed for their receptor need to be tweaked to suit the binding interactions with MPro. These compounds identified through the present study are functionally similar to linoleic acid, a free fatty acid that has been found to occupy the binding pocket of spike protein in SARS-CoV-2 [68]*.* Comparing the free fatty acid, linoleic acid, the GPR 120 agonists identified herein possess several beneficial physicochemical properties in terms of favourable log *P* values and topological polar surface area, making them suitable for oral administration (Fig. 2c).

Toxicity prediction for compound 34, using online tool “protox_II,” indicates that the molecule is safe for all the targets except showing carcinogenicity and hepatotoxicity of 0.51 and 0.64, respectively, further requiring structural modification. This compound also has an LD 50 value of 300 mg/kg and falls under predicted toxicity class 3, indicating it is only slightly toxic and slightly irritating.

## Conclusions

The present data supports the possibility of repurposing free fatty acid GPR 120 receptor agonists as potential inhibitors of Sars-COVID-19 M-Pro protein. Based on docking score and key interactions with the amino acid residues in the target protein, compound 34 could be used as a lead compound. The presence of COOH mimicking hydroxyl isoxazole group could provide necessary drug-like property in addition to maintaining H-bond and pi interactions with the receptor. Favorable logP and available physicochemical and toxicity data of compound 34 could shorten the drug development time to position the compound as an early lead candidate to overcome the hurdles in identifying therapeutic drugs in coronavirus infection.

## Supplementary Information

**Additional file 1.** Docking images of the inhibitors (ligands) on M^Pro^.

## Data Availability

Yes. Attached as Supporting information

## Abbreviations

COVID-19 MPro: Severe acute respiratory syndrome coronavirus 2 main protease
GPR-120 agonists: G-protein-coupled receptor or free fatty acid receptor-4 agonists
Docking: Phenyl propanoic acid
