# Supplementary material for: Molecular docking and identification of G-protein-coupled receptor 120 (GPR120) agonists as SARS COVID-19 MPro inhibitors
Source: J Genet Eng Biotechnol. 2022 Jul 18;20:108. doi: 10.1186/s43141-022-00375-8 (PMC9289937; doi:10.1186/s43141-022-00375-8)
Supplement: Supplementary file 1 — Additional file 1. Docking images of the inhibitors (ligands) on MPro. [file 43141_2022_375_MOESM1_ESM.docx]

Supplementary material for

**Molecular Docking and Identification of G Protein-coupled receptor 120 (GPR120) Agonists as SARS Covid 19 M^Pro^ Inhibitors**

Docking images of the inhibitors (ligands) on M^Pro^DHA (1)


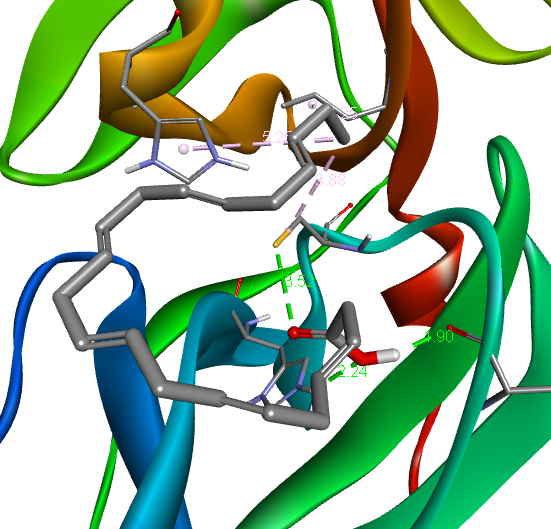

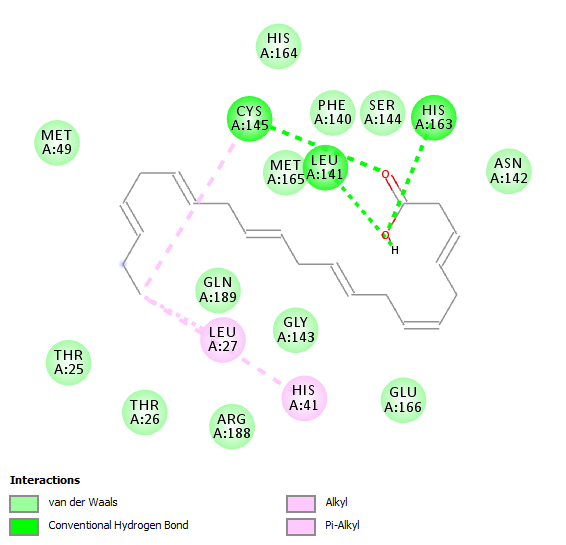


ALA (2)


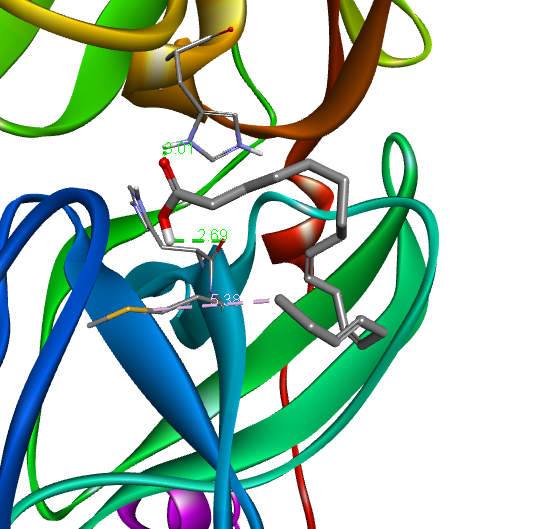

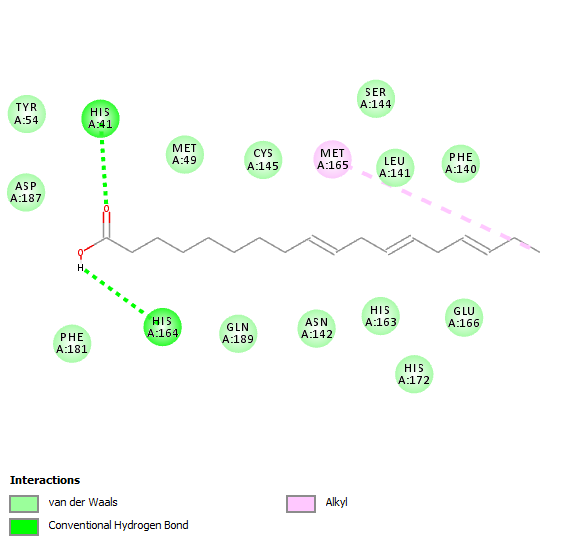


EPA (3)


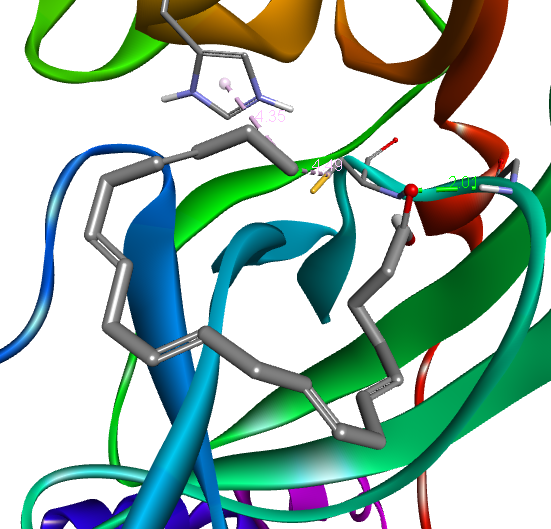

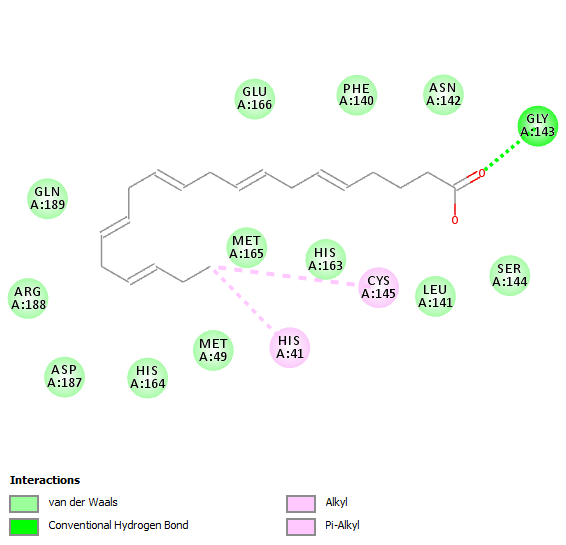


Grifolic acid (4)


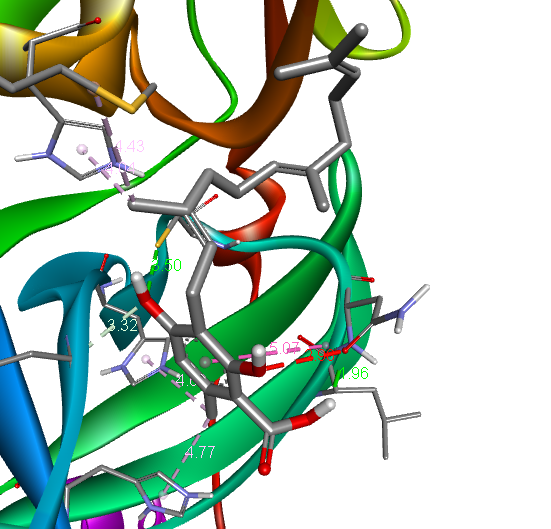

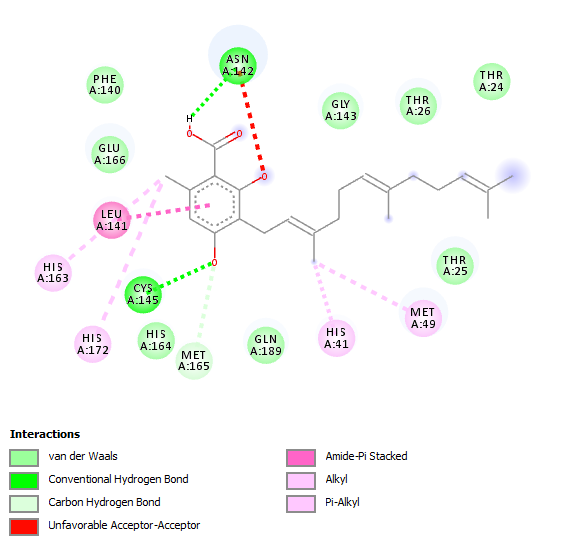


Grifolic acid methyl ether (5)


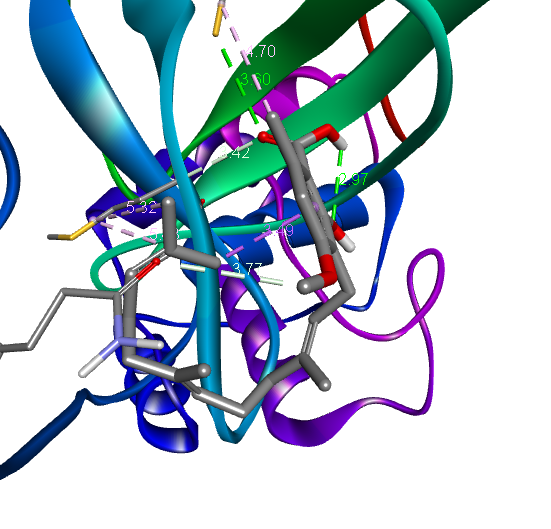

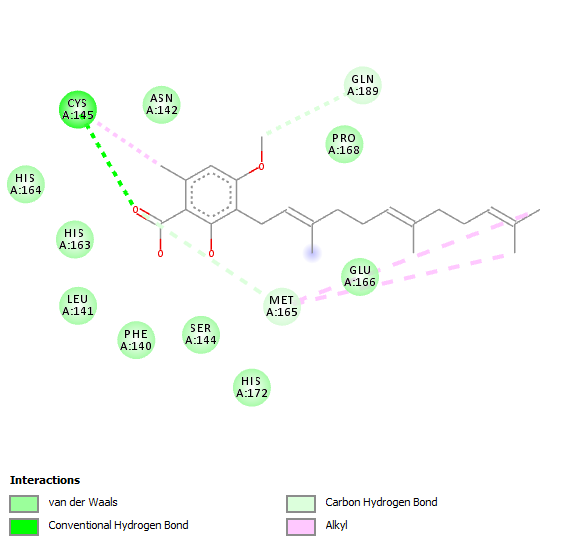


GW9508 (6)


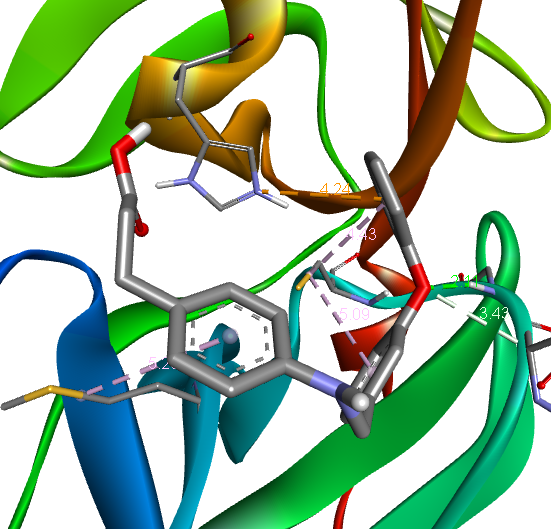

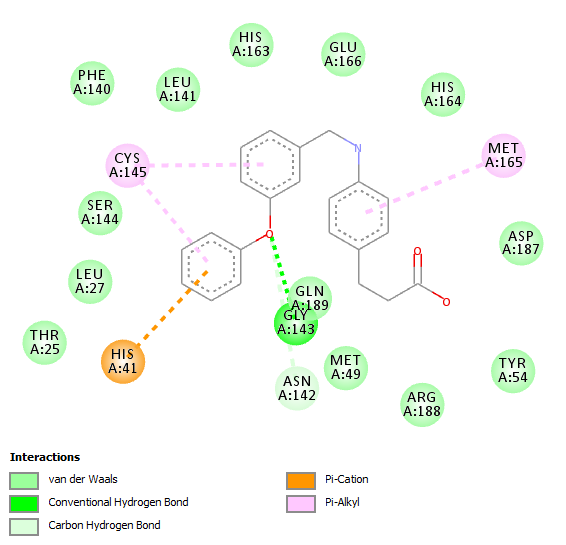


NCG21 (7)


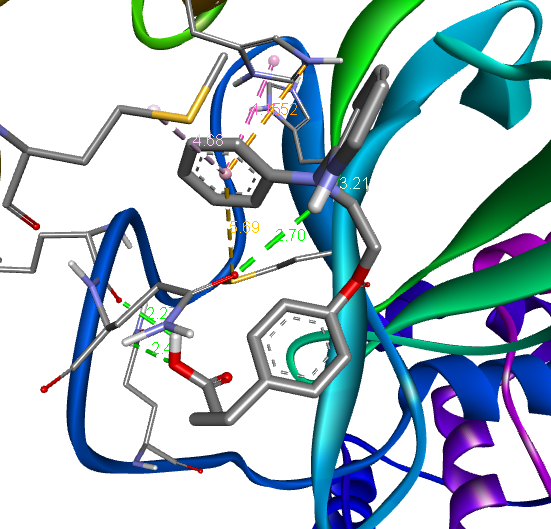

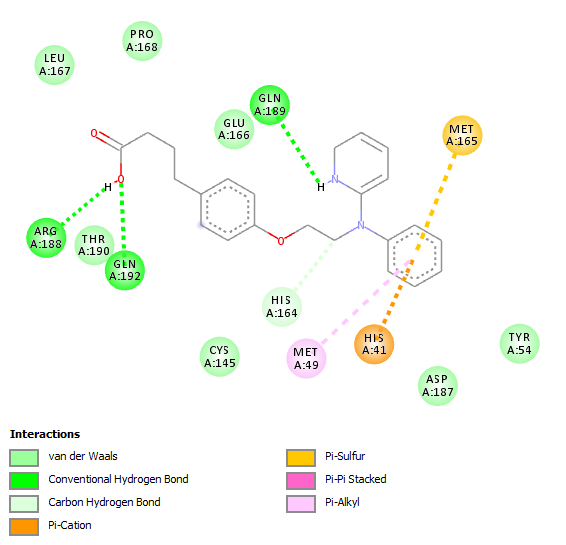


GSK137647A (8)


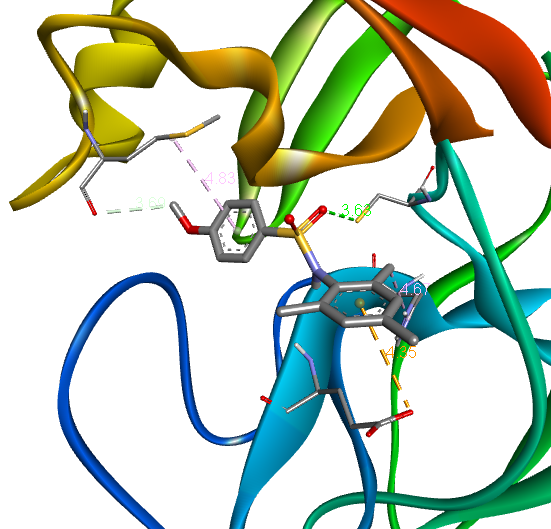

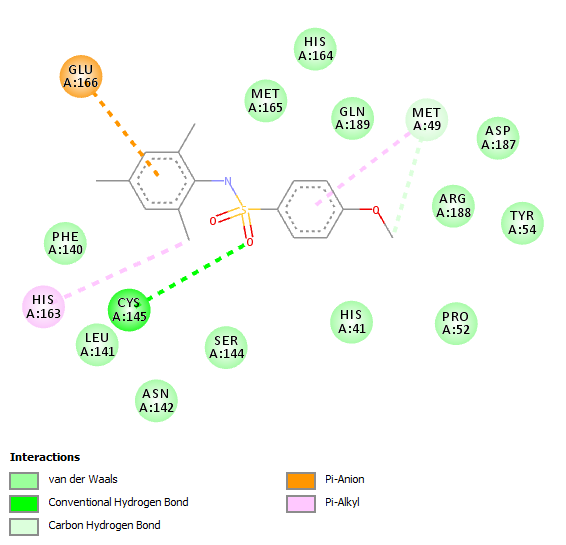


TUG891 (9)


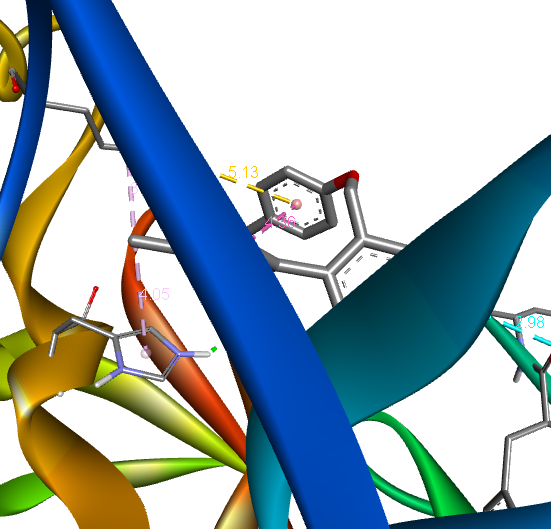

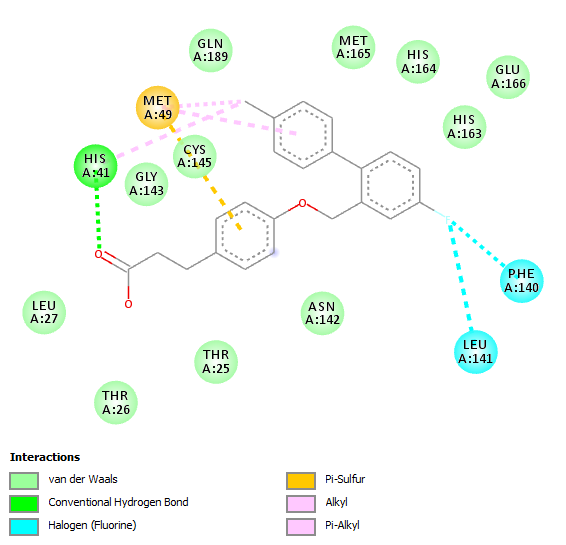


BiAr-PPA (10)


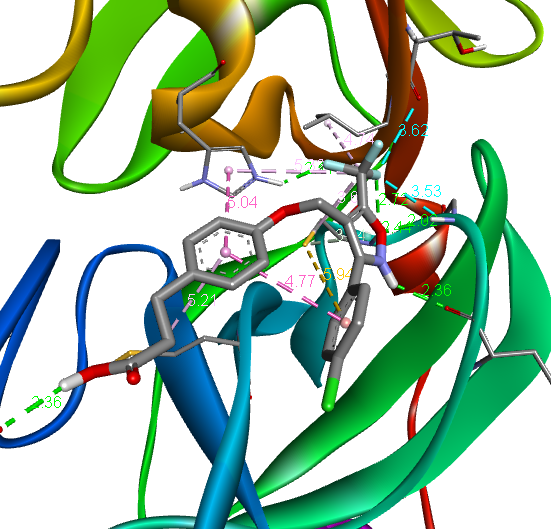

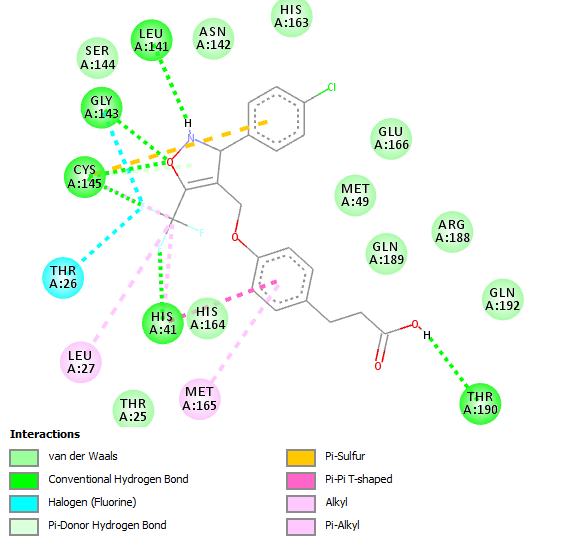


BiAr-PPA (11)


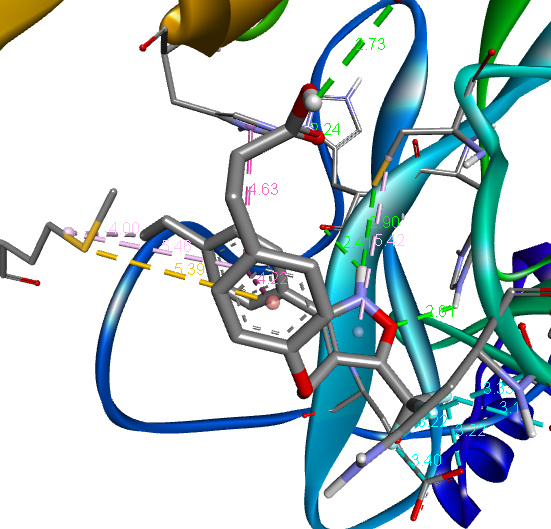

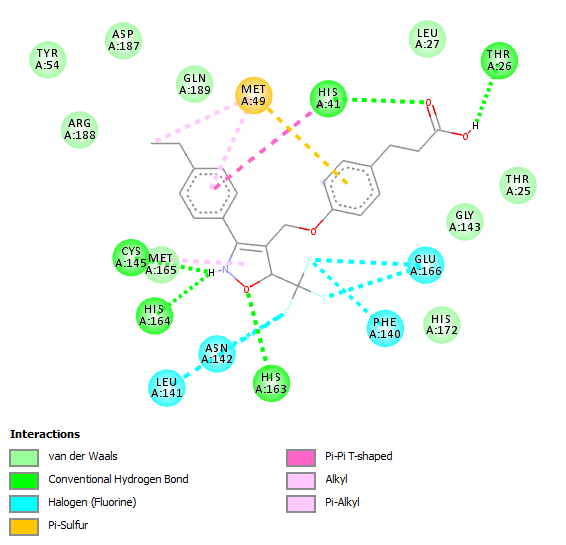


BiAr-PPA (12)


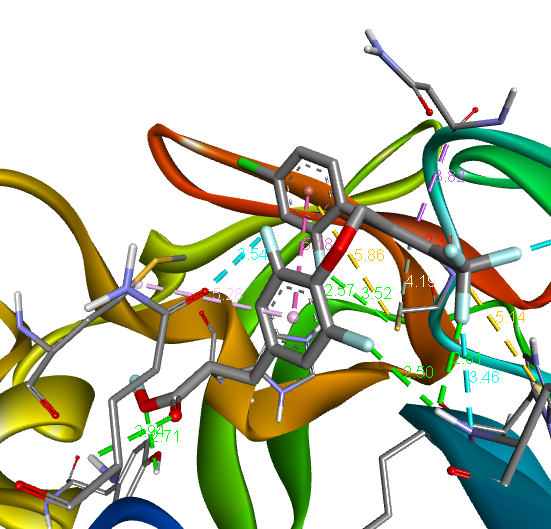

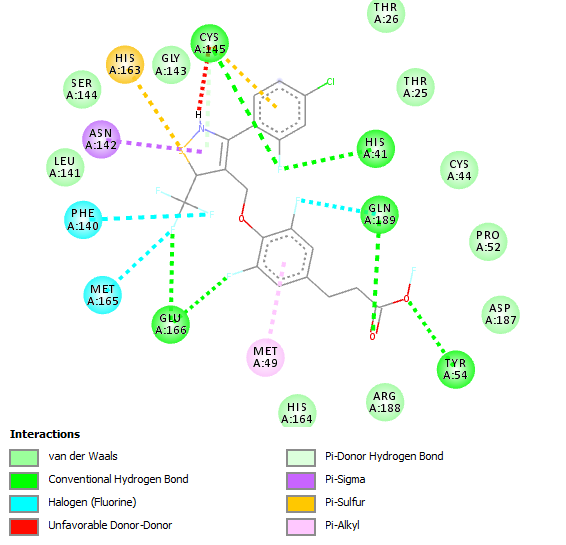


BiAr-PPA (13)


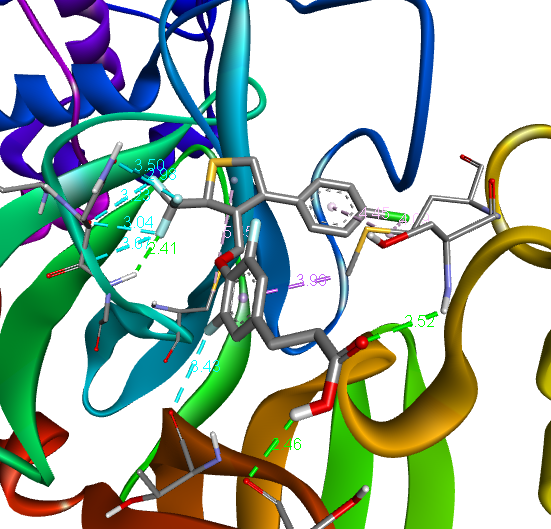

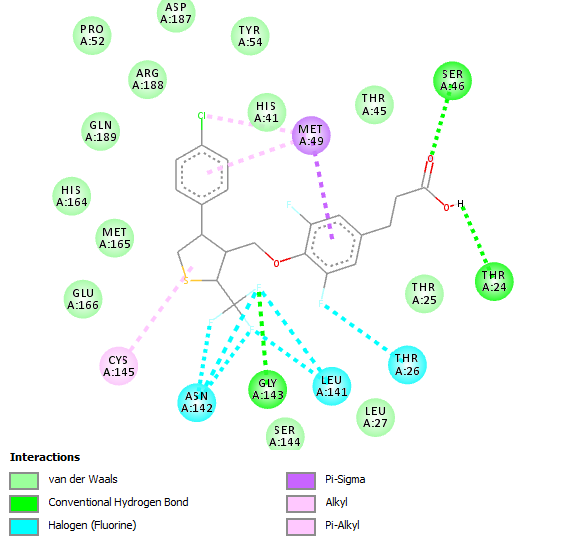


BiAr-PPA (14)


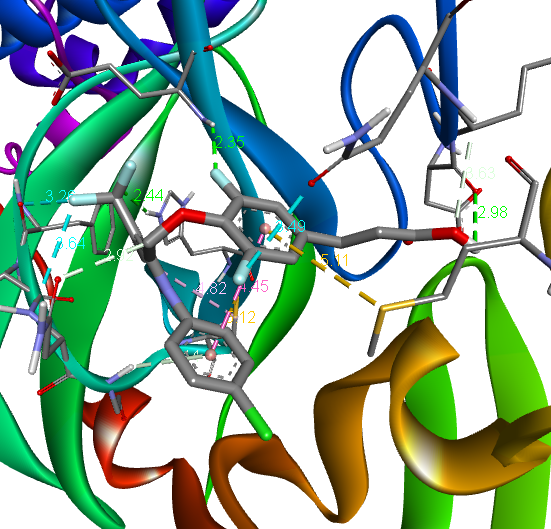

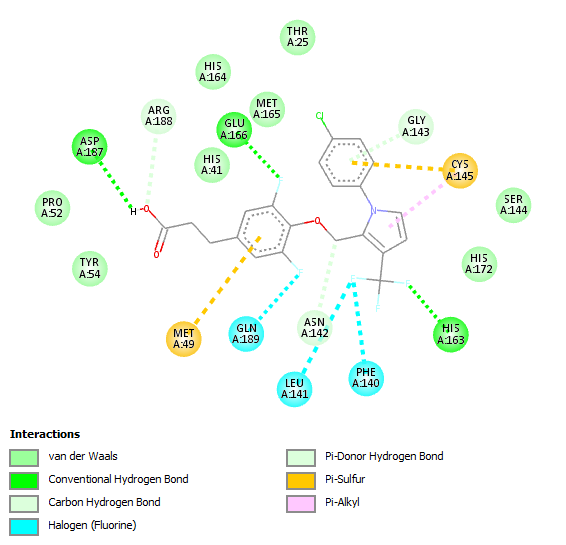


CycA_Hcyc--PPA (15)


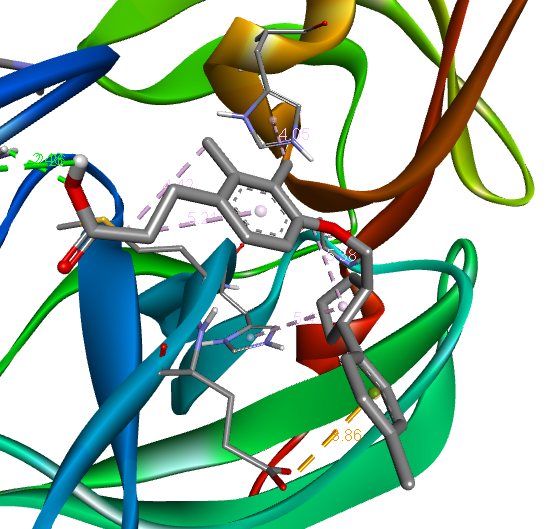

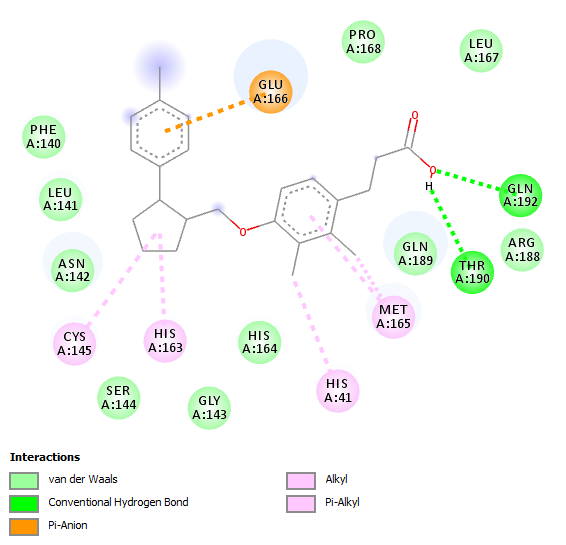


CycA_Hcyc--PPA (16)


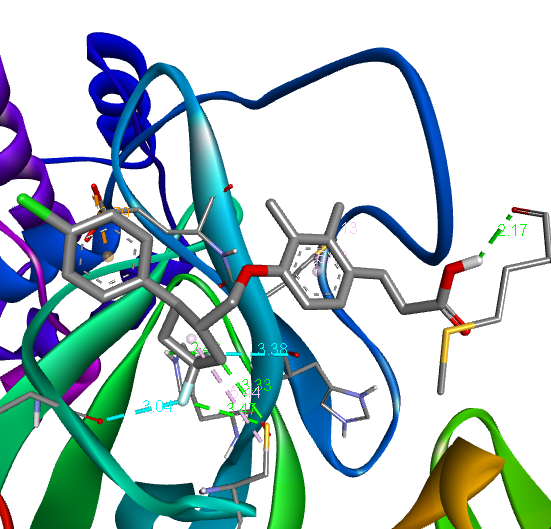

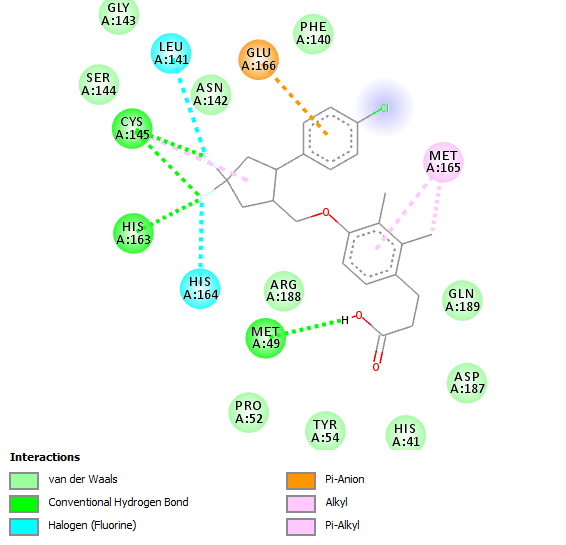


CycA_Hcyc--PPA (17)


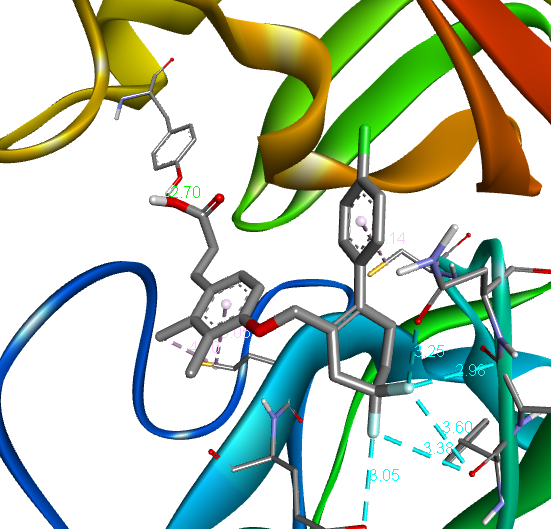

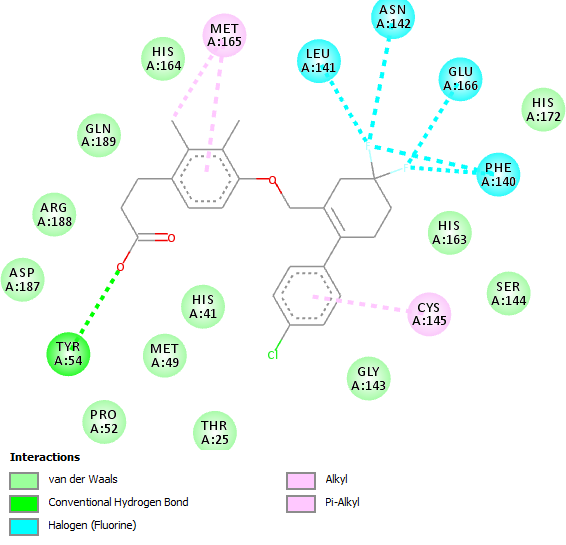


CycA_Hcyc--PPA (18)


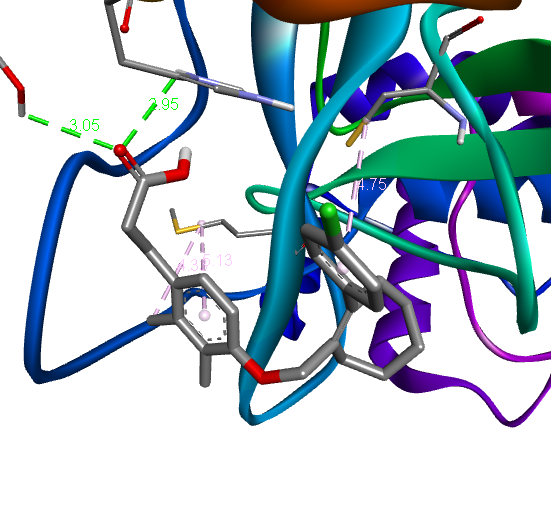

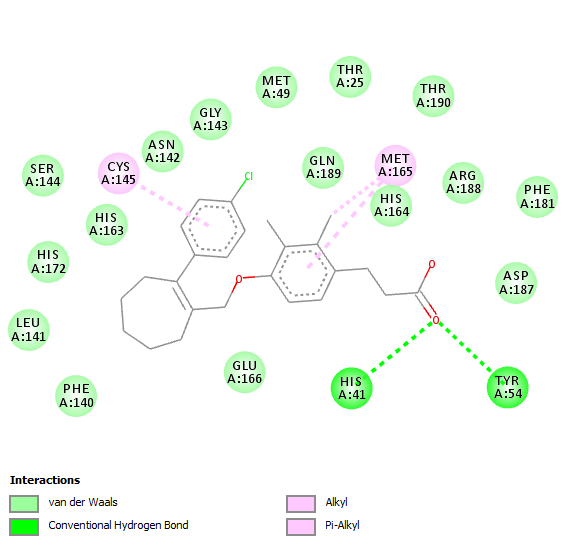


CycA_Hcyc--PPA (19)


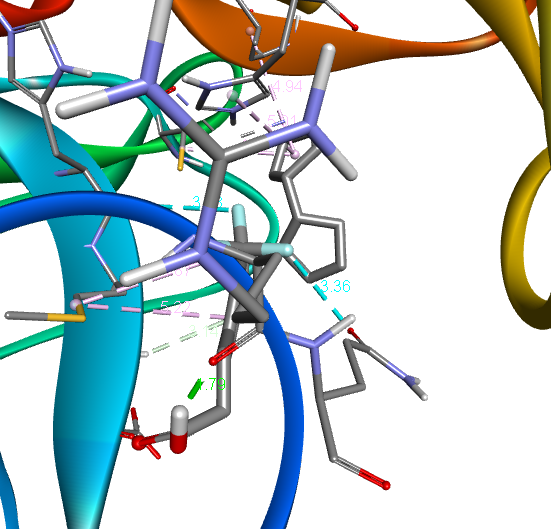

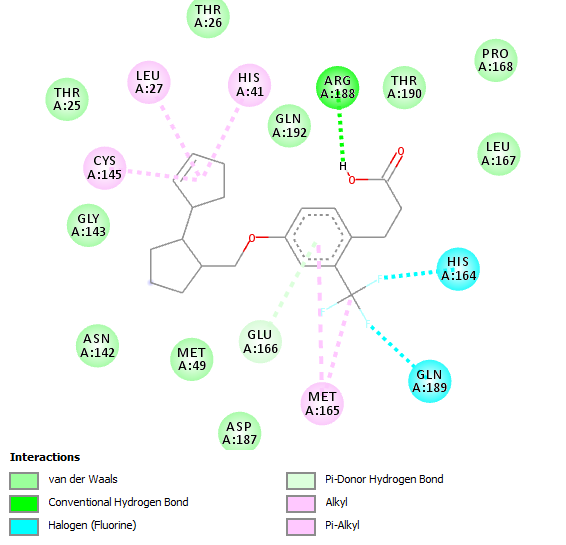


CycA_Hcyc--PPA (20)


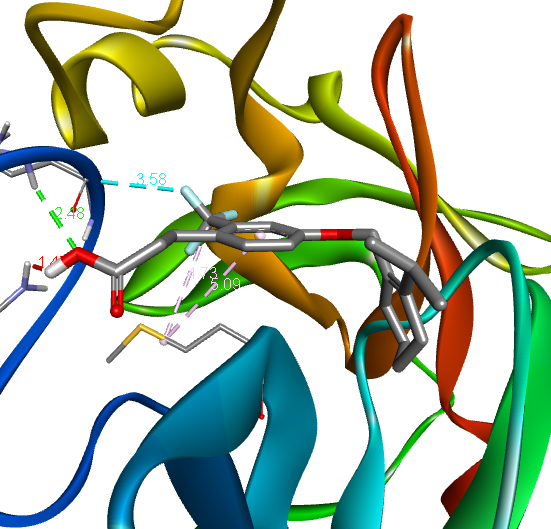

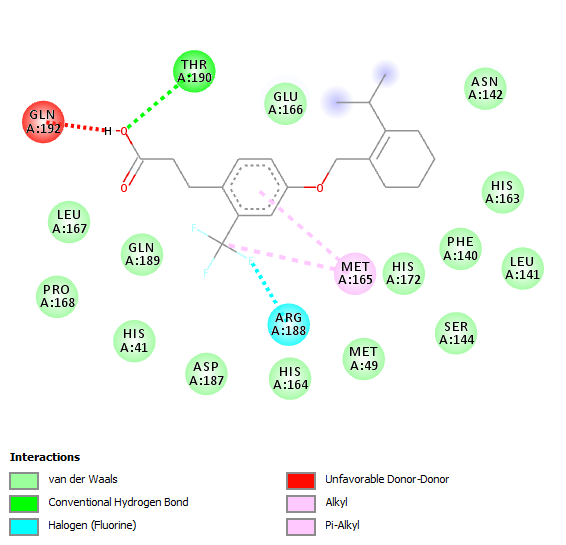


CycA_Hcyc--PPA (21)


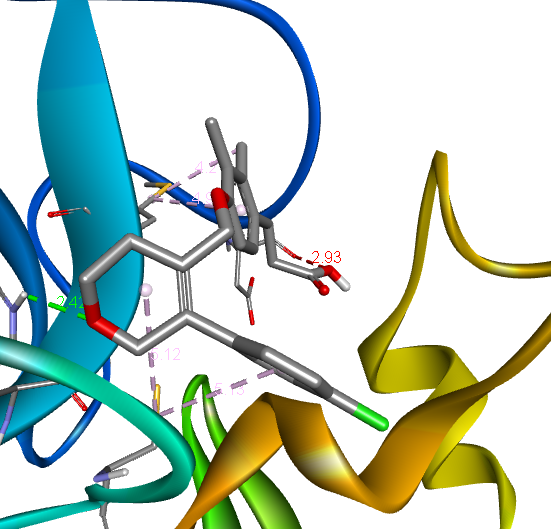

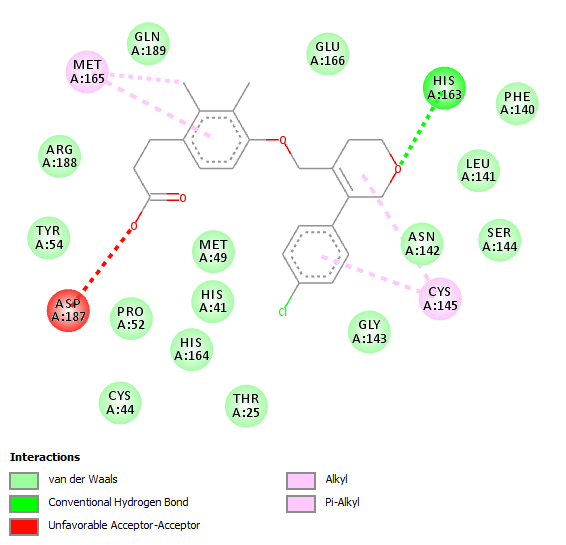


CycA_Hcyc--PPA (22)


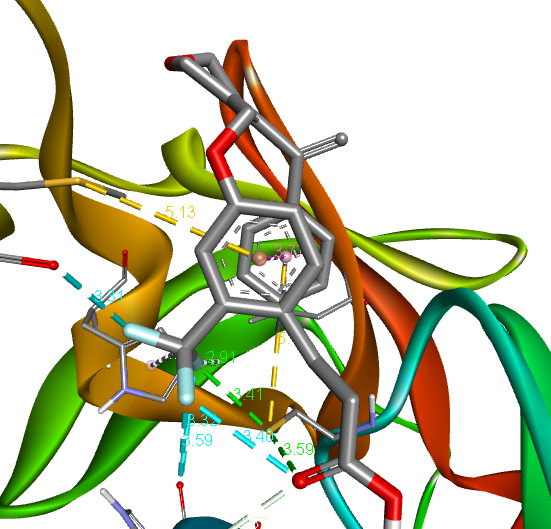

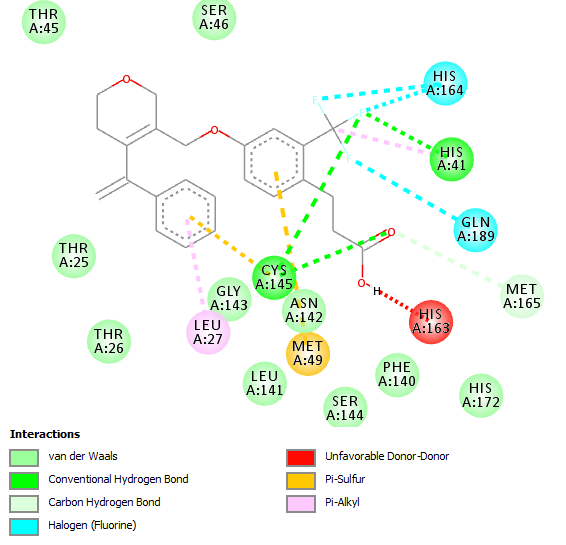


CycA_Hcyc--PPA (23)


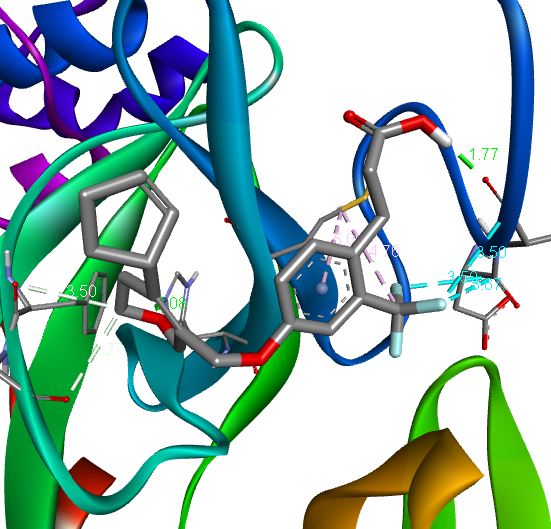

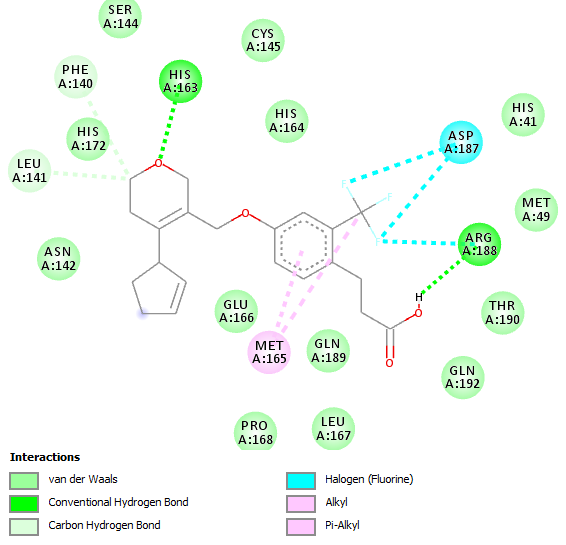


Metabolex (24)


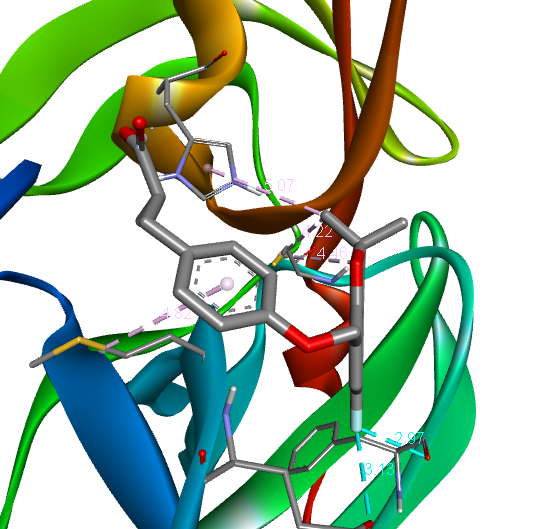

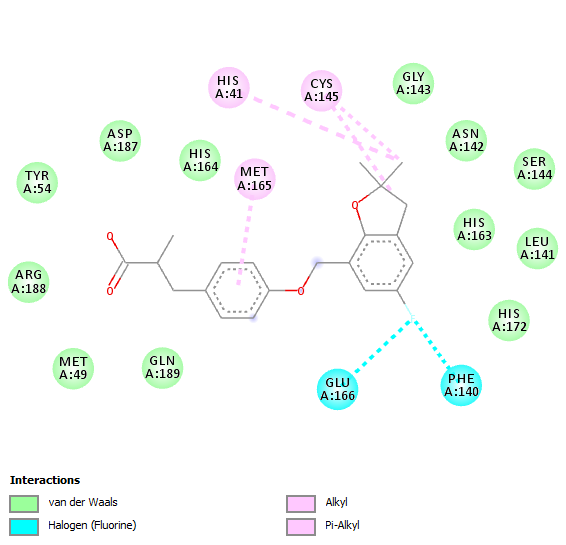


Janssen (25)


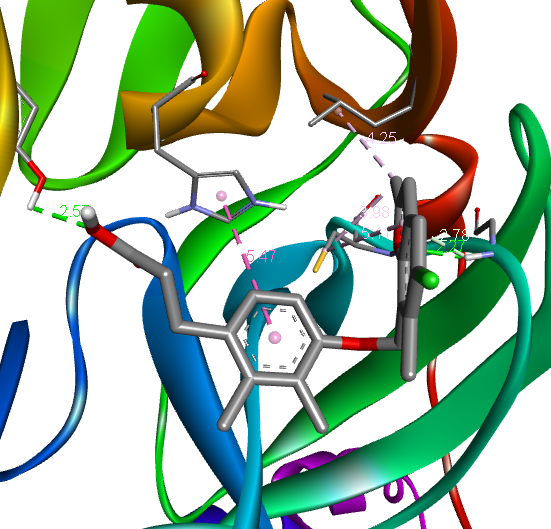

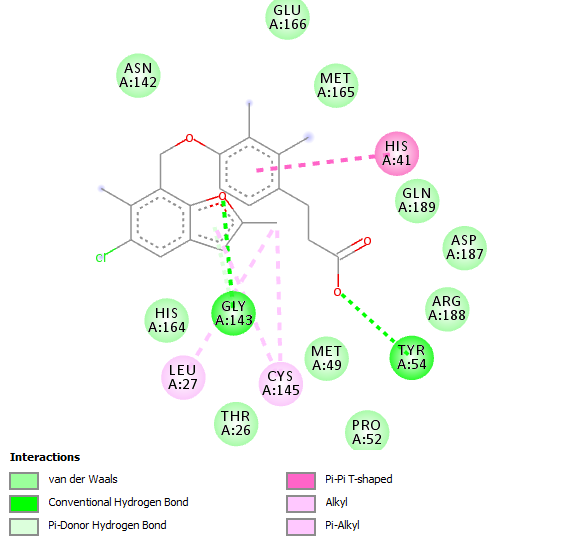


Janssen (26)


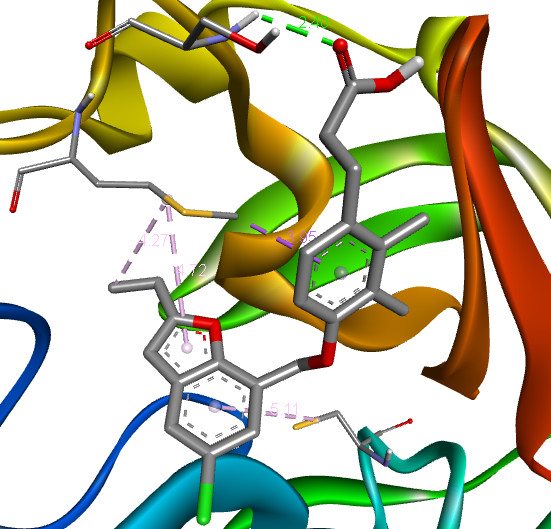

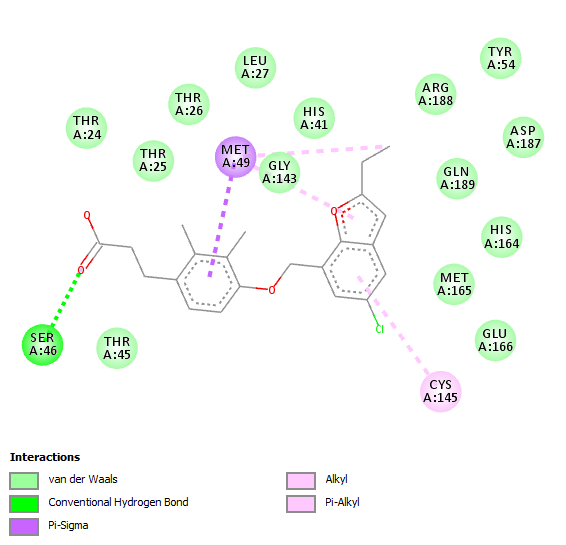


Janssen (27)


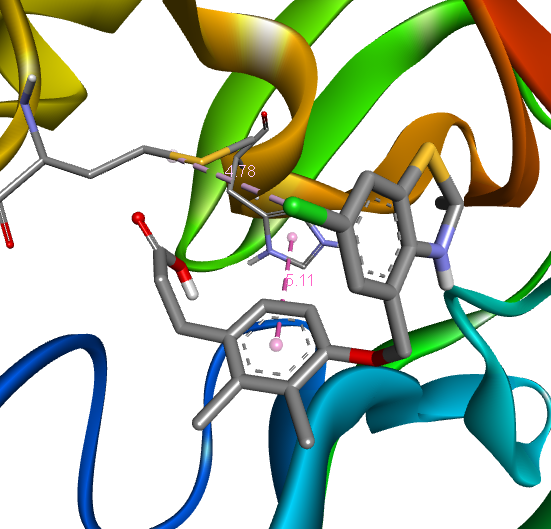

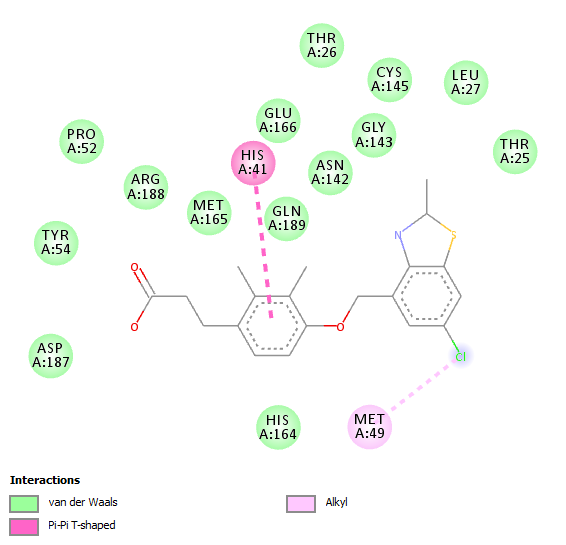


Merck (28)


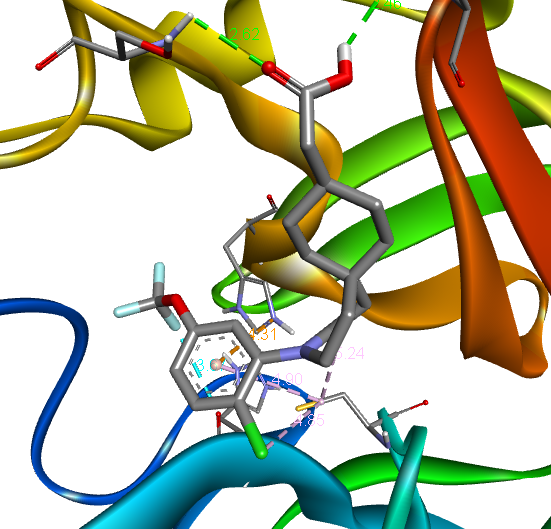

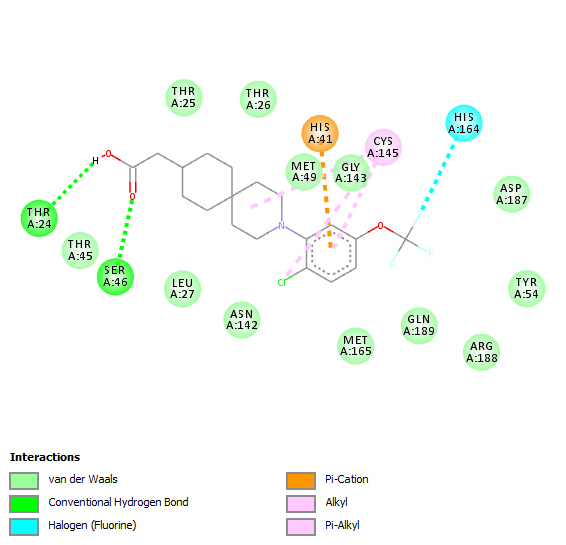


Merck (29)


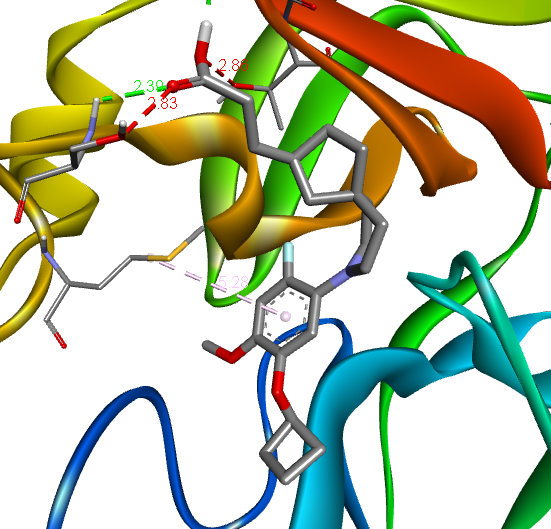

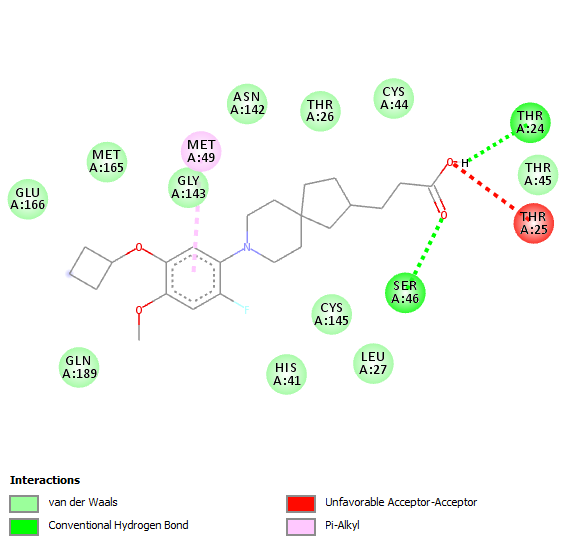


Merck (30)


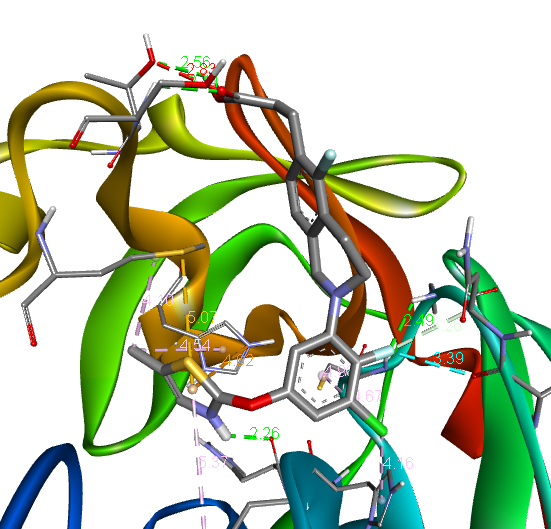

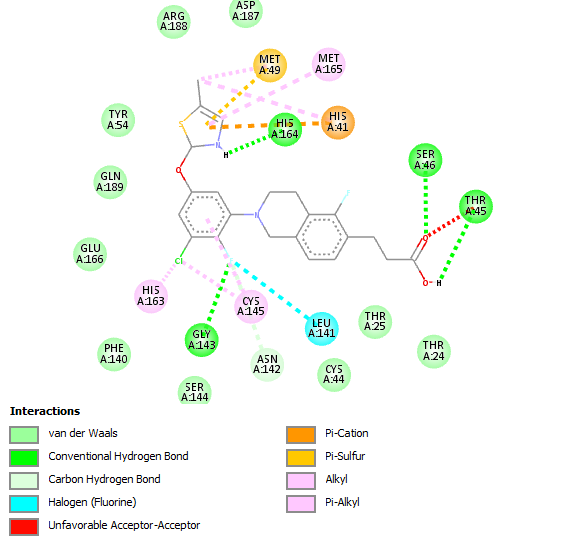


Merck (31)


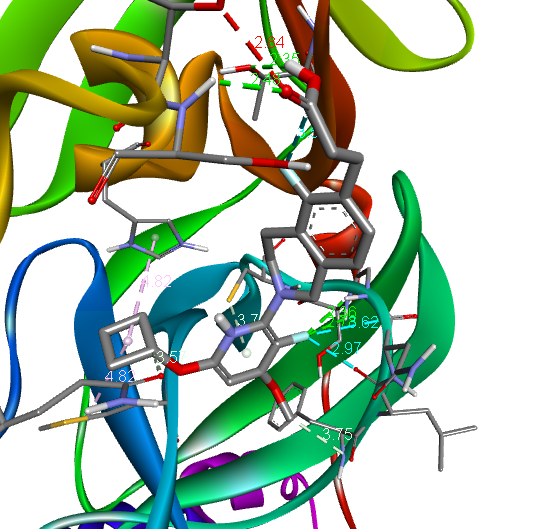

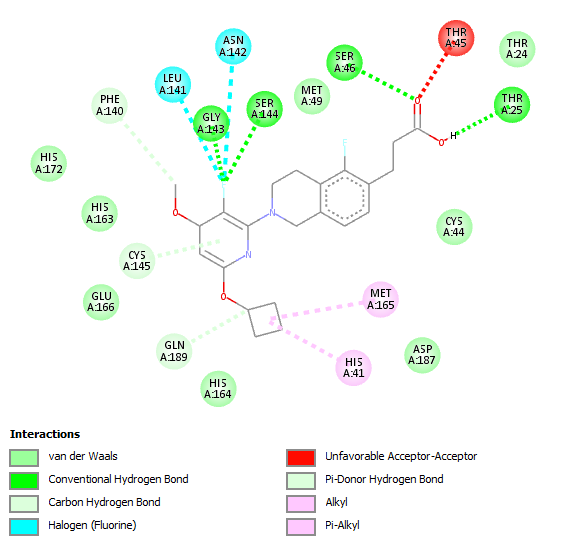


Merck (32)


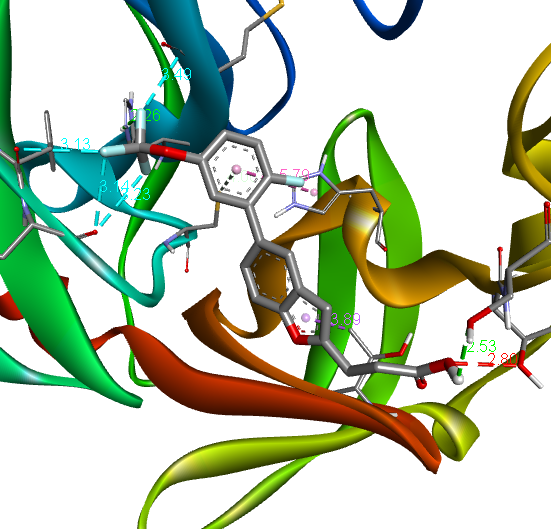

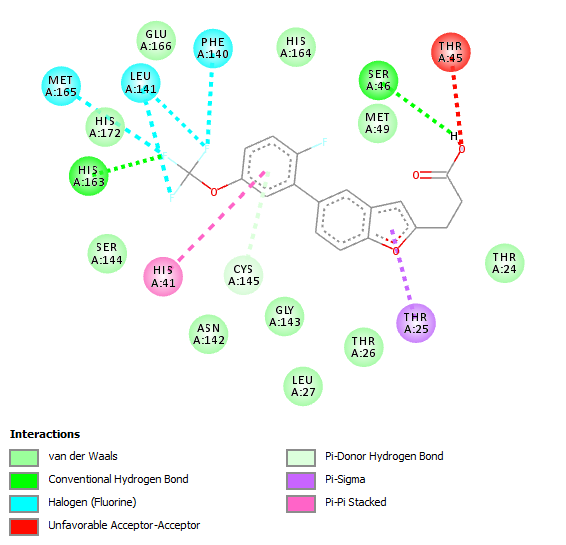


Merck (33)


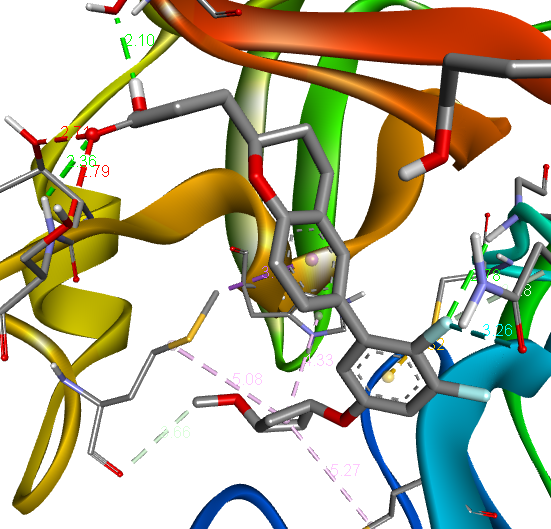

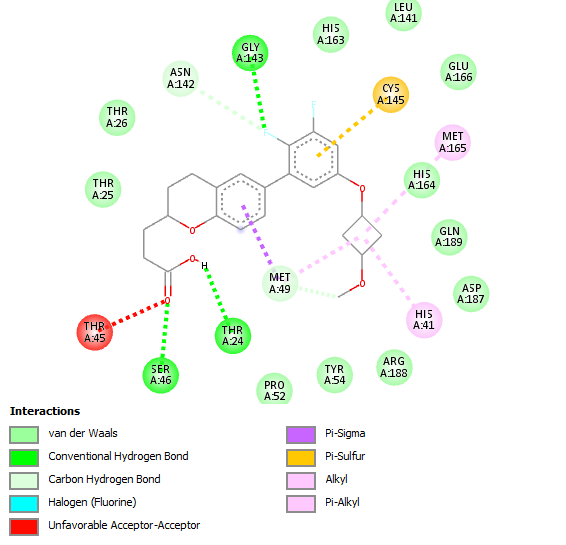


Merck (34)


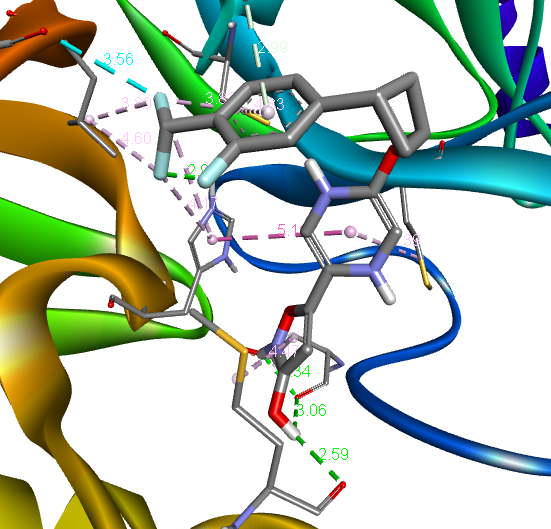

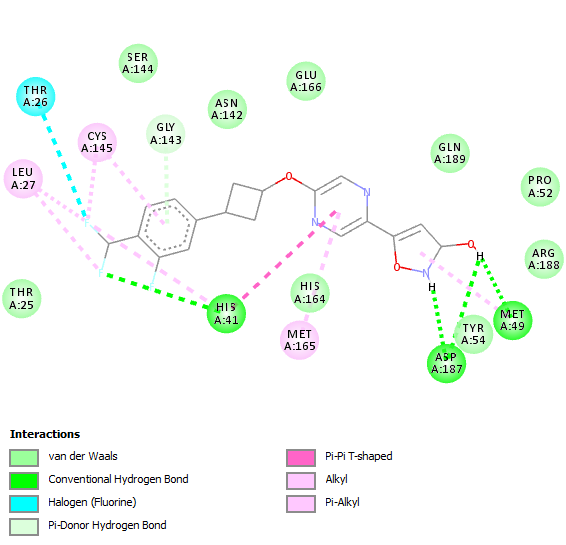


Merck (35)


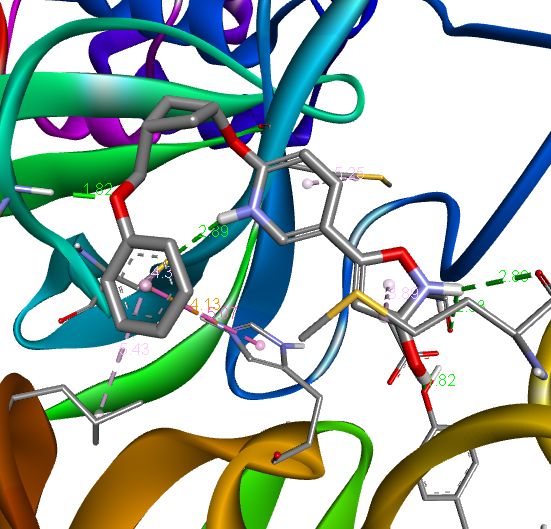

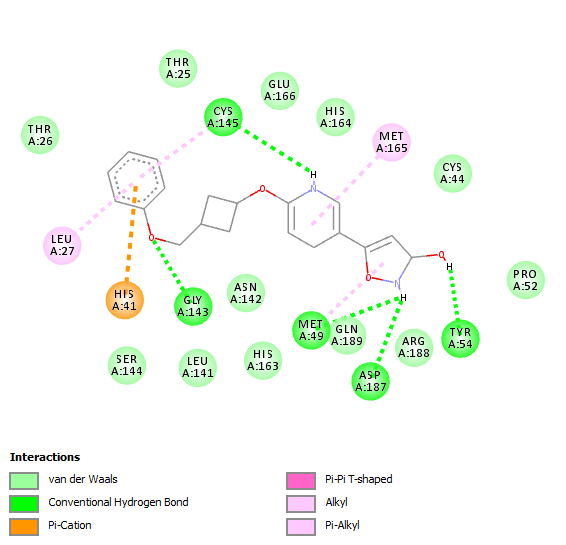


BMS (36)


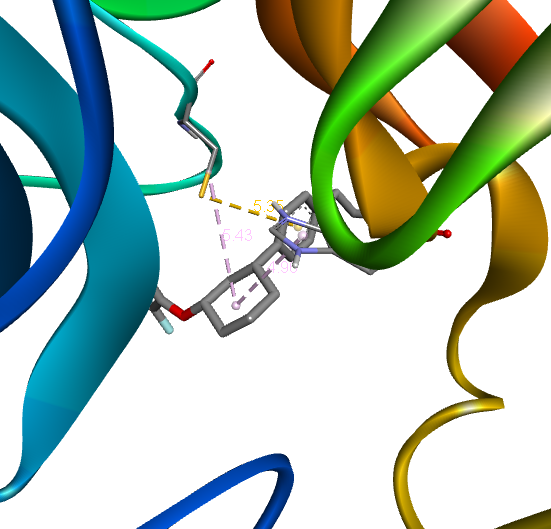

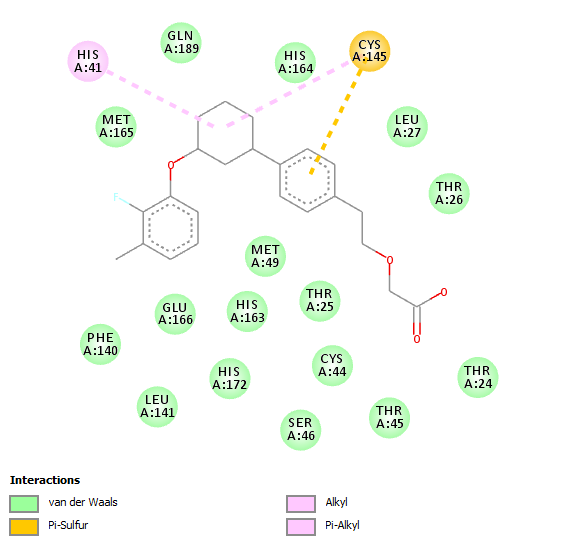


BMS (37)


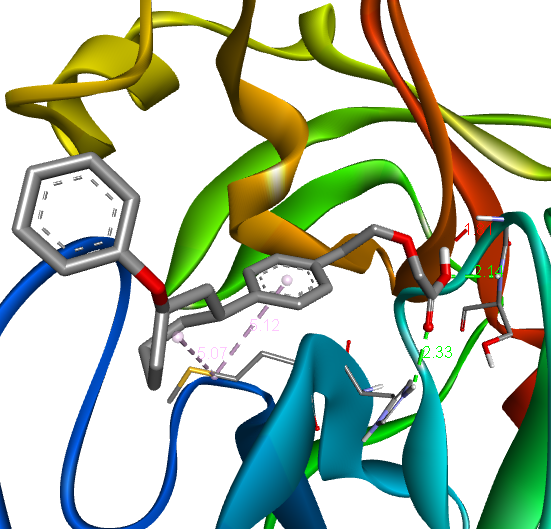

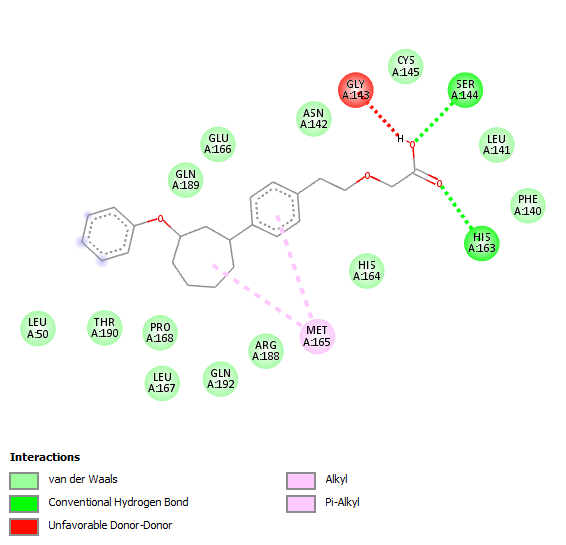


BMS (38)


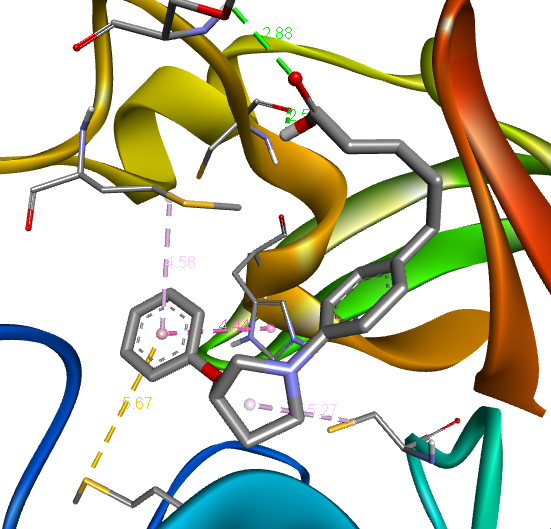

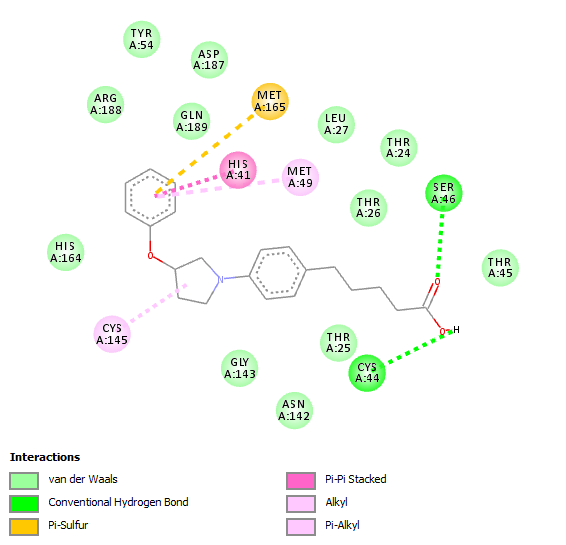


BMS (39)


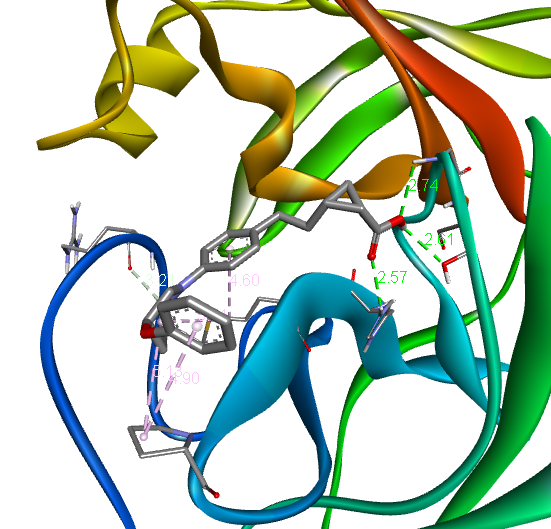

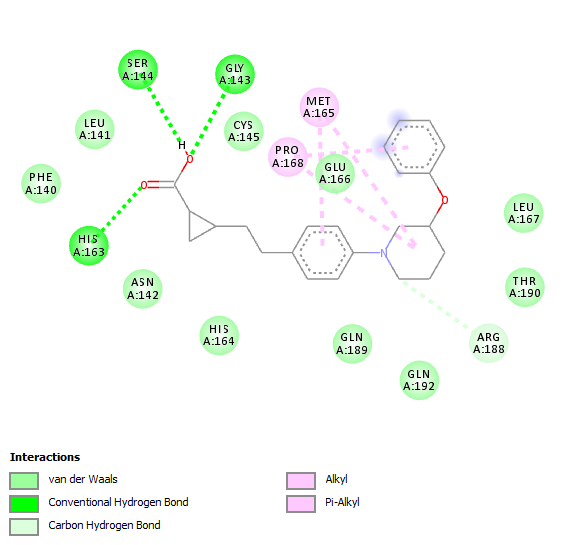


BMS (40)


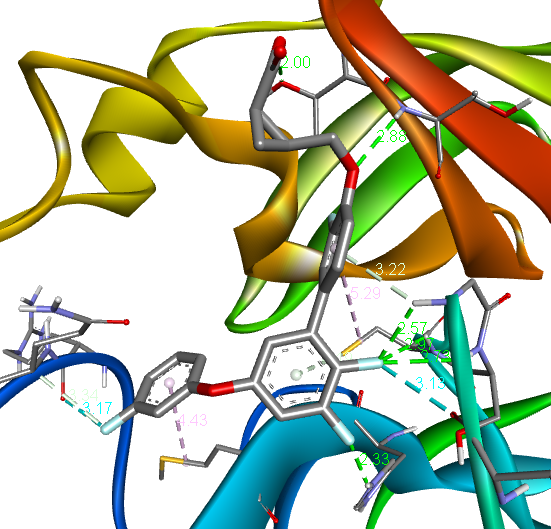

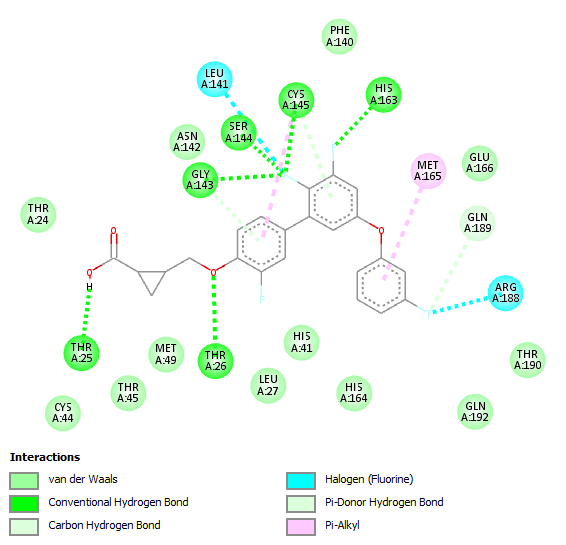


BMS (41)


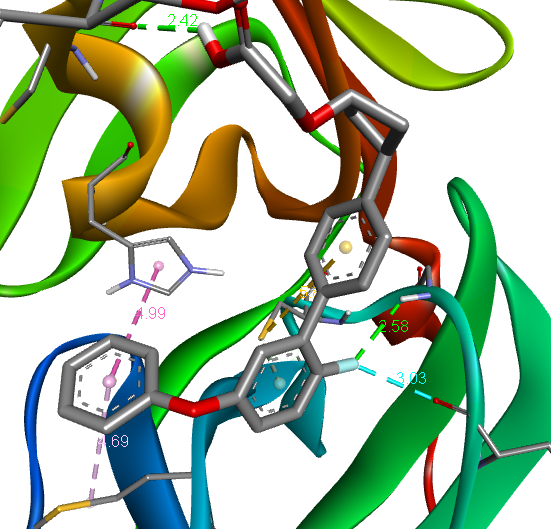

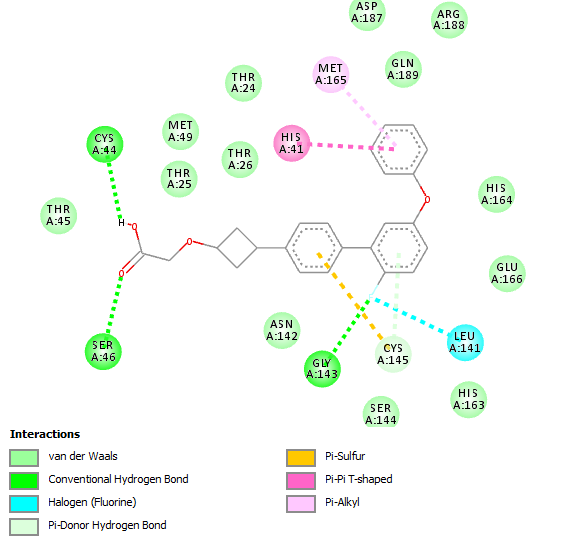


BMS (42)


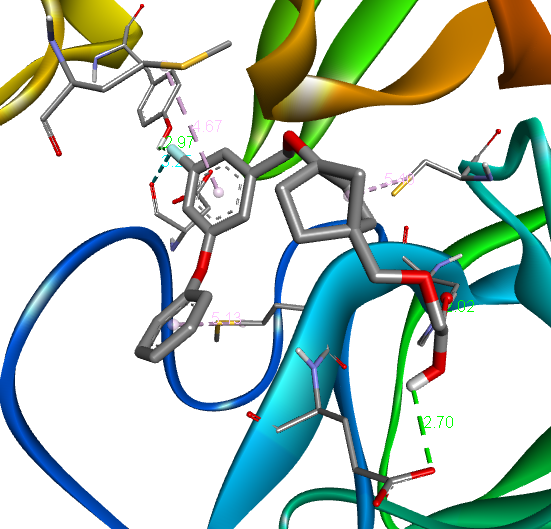

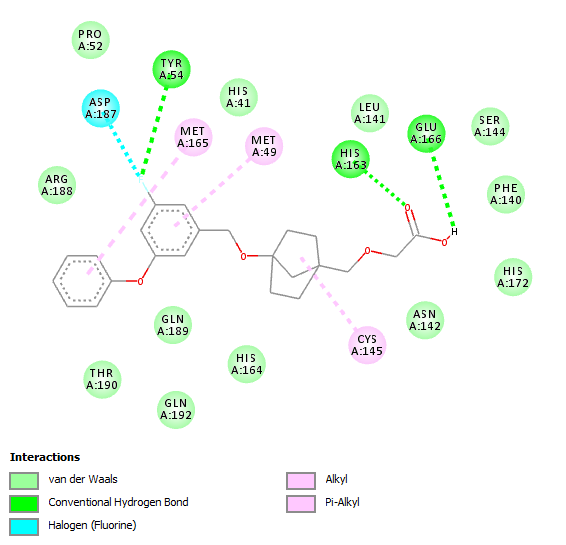


BMS (43)


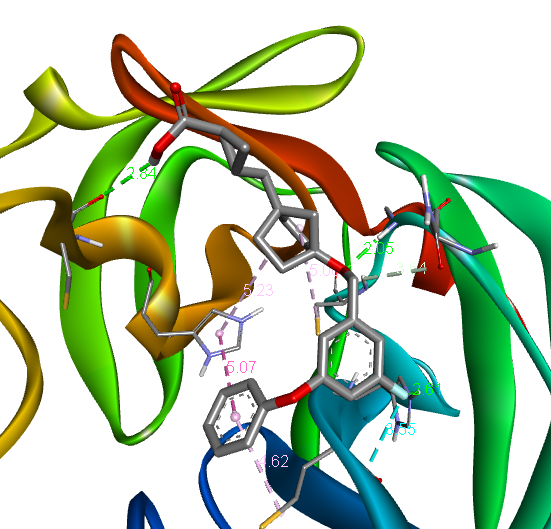

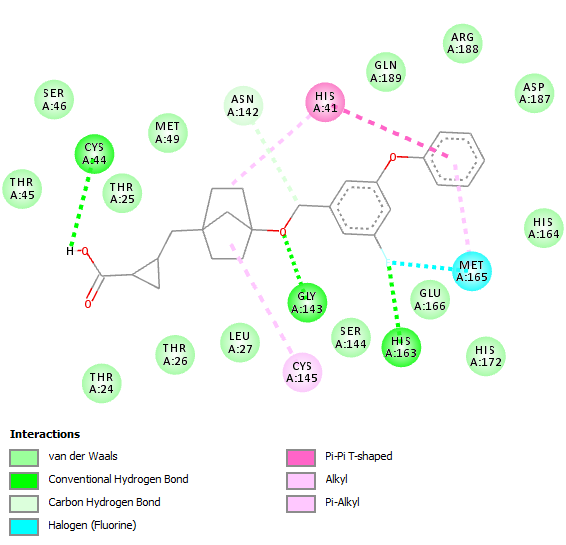


BMS (44)


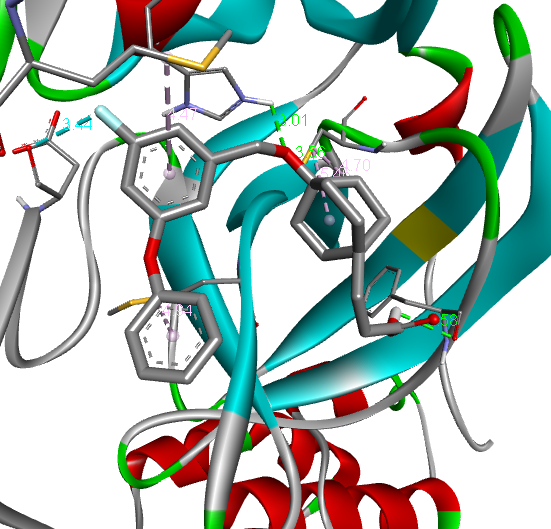

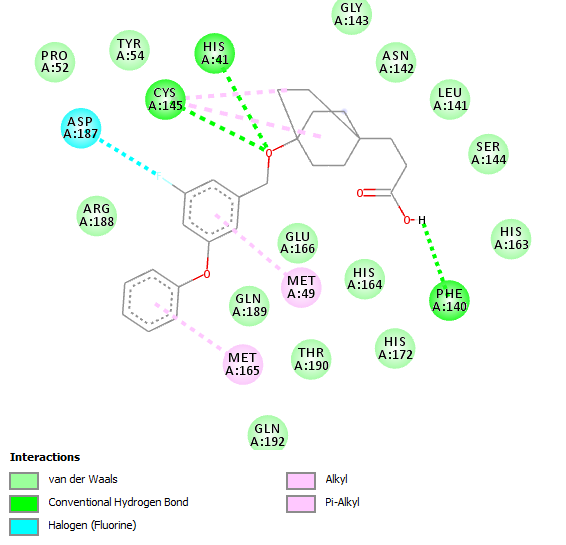


BMS (45)


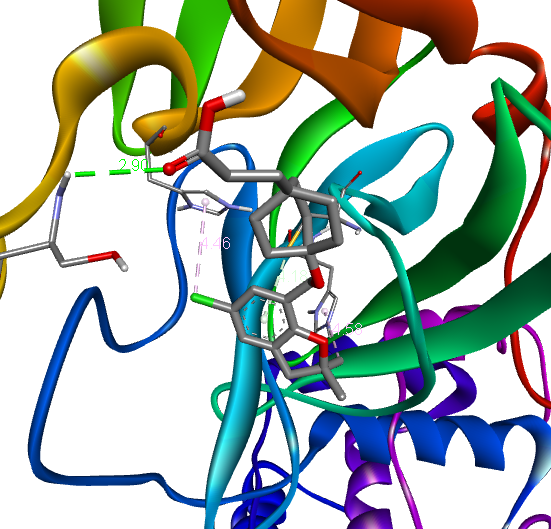

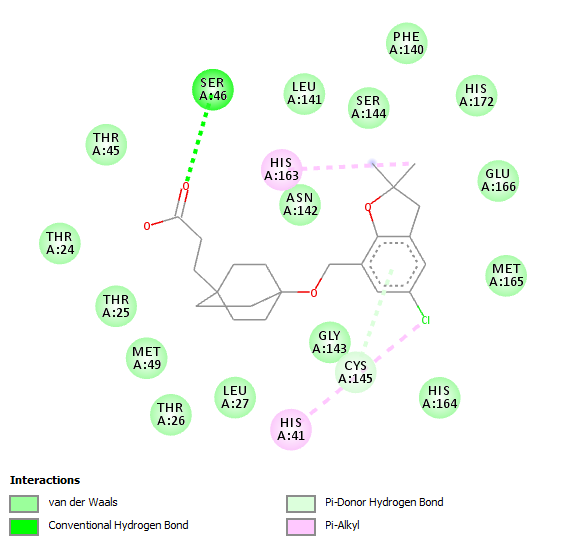


PML (46)


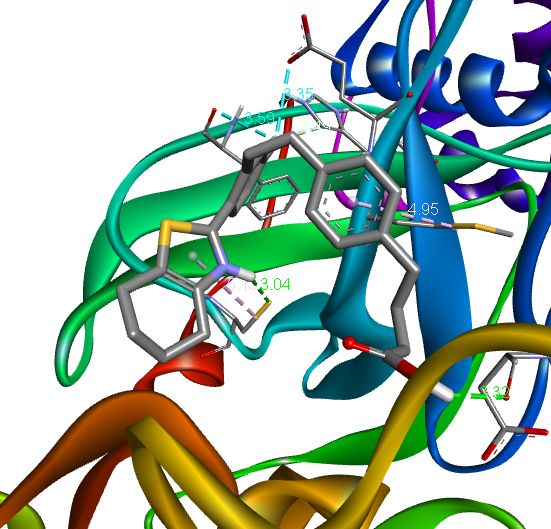

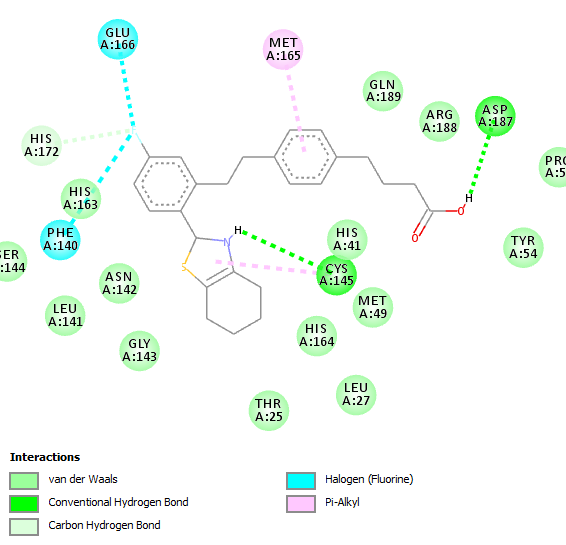


PML (47)


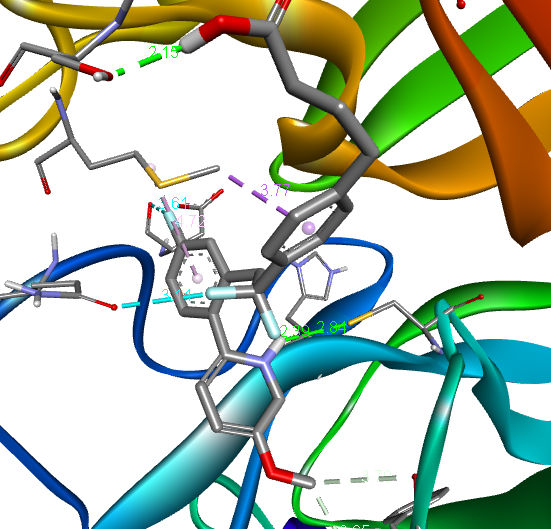

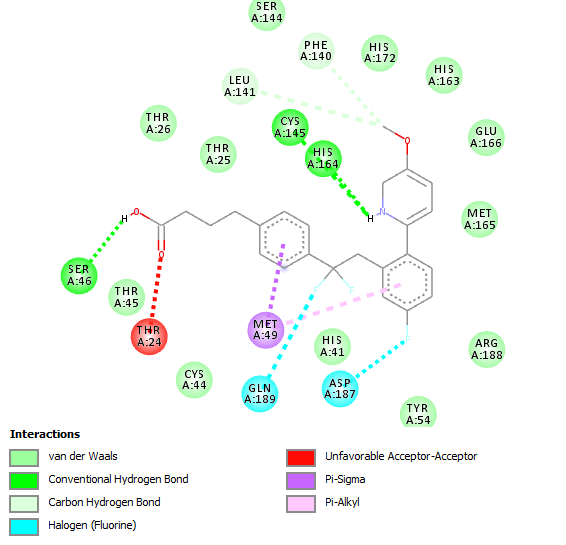


PML (48)


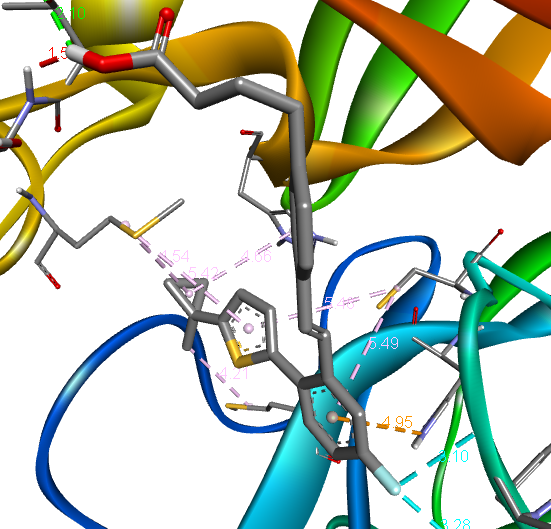

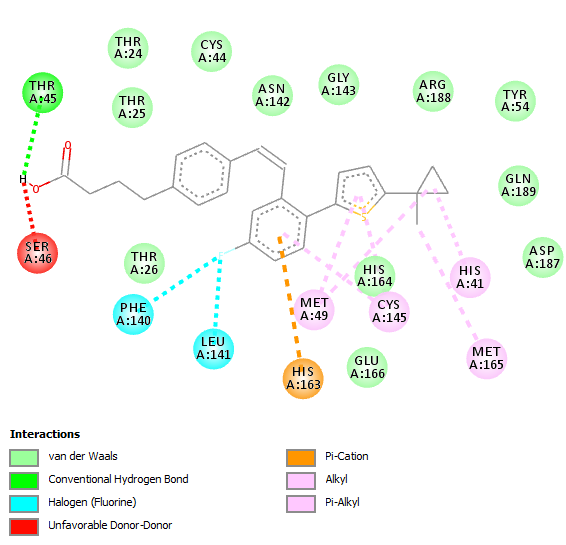


PML (49)


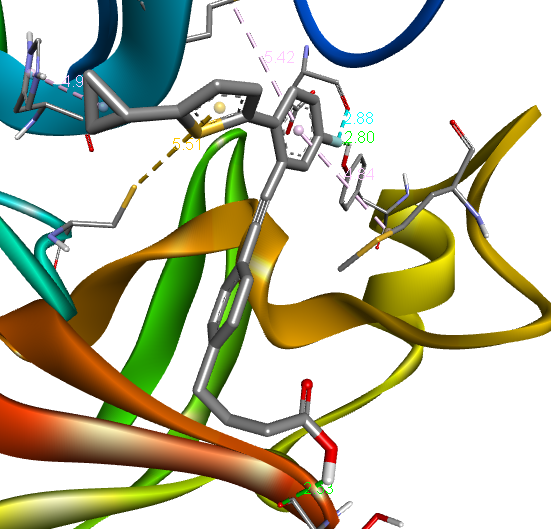

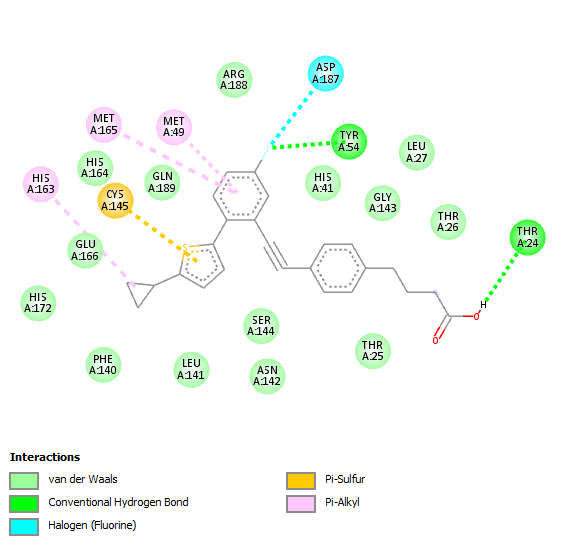


PML (50)


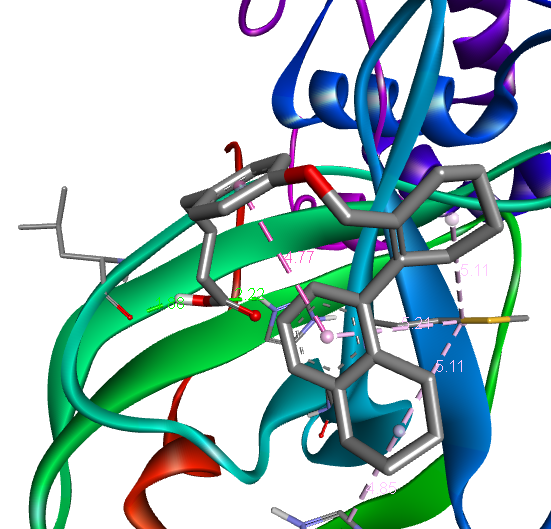

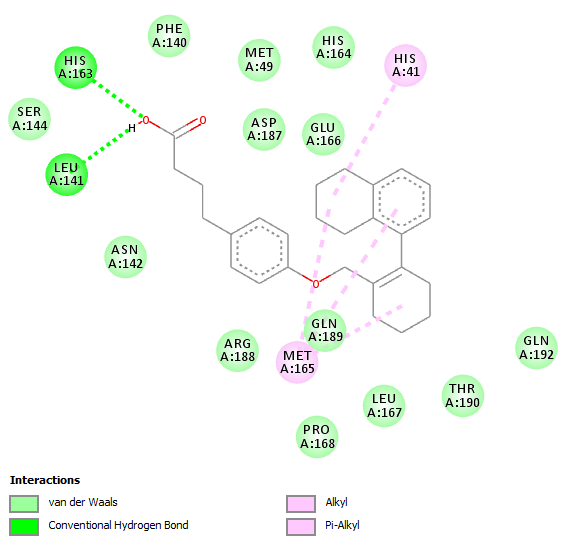


PML (51)

PML (52)

Calden (53)

Calden (54)

LG (55)

LG (56)

LG (57)

LG (58)

LG (59)

Ajinamoto (60)

DOMPE (61)

DOMPE (62)

AXXAM (63)

AXXAM (64)

GSK (65)

Uof B (66)

GSK (67)

ChPharmU (68)
